# Testing nature-based biopsychosocial resilience theory: a research programme protocol

**DOI:** 10.1186/s13690-026-01903-5

**Published:** 2026-04-24

**Authors:** Mathew P. White, Julia A. M. Egger, Giulia Amato, Thomas Astell-Burt, Stine Bekke-Hansen, Angel Borja, Angel Burov, Svea Busse, Caroline Costongs, Donka Dimitrova, Ilaria Doimo, Angel M. Dzhambov, Lewis R. Elliott, Alba Godfrey, James Grellier, Terry Hartig, Arnulf Hartl, Patrik Karlsson Nyed, Cecil Konijnendijk, Melissa Lem, Jill S. Litt, Rebecca Lovell, Freddie Lymeus, Aynur Mammadova, Leanne Martin, Angelica Moè, Sarah Morgan Trimmer, Colm O’Driscoll, Johan Östberg, Sabine Pahl, Francesca Pazzaglia, Christina Pichler, Alexandria Poole, Sarai Pouso, Nooshin Razani, Todora Rogelja, Laura Secco, Ulrika K. Stigsdotter, Sus Sola Corazon, Georgina Sowman, Maria C. Uyarra, Agnes E. van den Berg, Thomas van Rompay, Martin Voracek, Nancy M. Wells, Benedict W. Wheeler, Matilda van den Bosch

**Affiliations:** 1 Department of Clinical and Health Psychology, Faculty of Psychology, University of Vienna, Wächtergasse 1, Vienna, A-1090 Austria; 2 Vienna Cognitive Science Hub, University of Vienna, Vienna, Austria; 3 Environment and Climate Research Hub, University of Vienna, Vienna, Austria; 4 European Centre for Environment & Human Health, University of Exeter, Exeter, UK; 5 ETIFOR, Padua, Italy; 6 Centre for Flourishing Cities, School of Architecture, Design and Planning, University of Sydney, Sydney, New South Wales Australia; 7 Westmead Applied Research Centre, Westmead Hospital, Sydney, New South Wales Australia; 8 Charles Perkins Centre, University of Sydney, Sydney, New South Wales Australia; 9 Net Zero Institute, University of Sydney, Sydney, New South Wales Australia; 10 Population Wellbeing and Environment Research Lab (PowerLab), Sydney, New South Wales Australia; 11 Department of Geosciences and Natural Resource Management, University of Copenhagen, Copenhagen, Denmark; 12 AZTI, Marine Research, Basque Research and Technology Alliance (BRTA), Pasaia, Spain; 13 Environmental Health Division, Research Institute at Medical University of Plovdiv, Medical University of Plovdiv, Plovdiv, Bulgaria; 14 Department of Urban Planning, Faculty of Architecture, University of Architecture, Civil Engineering and Geodesy, Sofia, Bulgaria; 15 ISGlobal, Barcelona, Spain; 16 Universitat Pompeu Fabra, Barcelona, Spain; 17 Ciber on Epidemiology and Public Health (CIBERESP), Madrid, Spain; 18 EuroHealthNet, Brussels, Belgium; 19 Department of Health Management and Health Economics, Faculty of Public Health, Medical University of Plovdiv, Plovdiv, Bulgaria; 20 Institute for Housing and Urban Research, Uppsala University, Uppsala, Sweden; 21 Department of Psychology, Uppsala University, Uppsala, Sweden; 22 Institute of Ecomedicine, Paracelsus Medical University, Salzburg, Austria; 23 Nature Based Solutions Institute, Malmö, Sweden; 24 Department of Family Practice, University of British Columbia, Vancouver, Canada; 25 Department of Land, Environment, Agriculture & Forestry (TESAF), University of Padua, Padua, Italy; 26 Department of General Psychology, University of Padua, Padua, Italy; 27 Primary Care, Population Science and Medical Education, University of Southampton, Southampton, UK; 28 Swedish University of Agricultural Sciences, Alnarp, Sweden; 29 Environmental Psychology Group, Department of Cognition, Emotion, and Methods in Psychology, Faculty of Psychology, University of Vienna, Vienna, Austria; 30 Philosophy Section, Behavioral, Management and Social Sciences Faculty, University of Twente, Twente, Netherlands; 31 University of California San Francisco, San Francisco, California USA; 32 Advanced Wellbeing Research Centre, Sheffield Hallam University, Sheffield, UK; 33 Department of Communication Science, University of Twente, Enschede, the Netherlands; 34 Department of Cognition, Emotion, and Methods in Psychology, Faculty of Psychology, University of Vienna, Vienna, Austria; 35 Department of Human Centered Design, College of Human Ecology, Cornell University, Ithaca, New York USA; 36 School of Population and Public Health, University of British Columbia, Vancouver, Canada; 37 European Forest Institute, Biocities facility, Rome, Italy

**Keywords:** Biopsychosocial resilience, blue/green spaces, Complex interventions, Longitudinal cohort studies, Mindfulness, Nature-based therapies, Social innovation actions, Social-ecological resilience, Health-related quality of life, Randomised controlled trials, Well-being

## Abstract

**Background:**

Interventions that use nature contact to promote health and well-being exist at the societal/infrastructural level (incl. nature-based solutions) and the individual/group level (incl. nature-based therapies). One way nature-based therapies promote health is by fostering resilience to manage stressors. Nature-based biopsychosocial resilience theory (NBRT) provides a framework to explain how nature plays a role in how resilience-related adaptive resources are built and maintained but this has yet to be tested.

**Methods:**

The current paper outlines a four-year multi-country (Austria, Belgium, Bulgaria, Denmark, Italy, the Netherlands, Spain, Sweden, the UK) research programme that tests the NBRT framework and explores how nature-based therapies can build and maintain individual and community resilience. As well as reviewing and mapping existing interventions globally, nine case studies across Europe are exploring how nature promotes resilience across: (1) whole populations (three case studies); (2) individuals at-risk of metabolic syndrome (three case studies); and (3) individuals with existing issues such as chronic stress, mobility challenges, or cognitive impairments (three case studies). Three case studies use longitudinal cohorts, five use randomised controlled trials, and one a practitioner shared-experience approach. The determinants and impacts of nature-based therapies are also considered beyond effects on individual participants, by assessing distributional issues (e.g., health equity), environmental impacts, financial implications, broader societal acceptability and engagement, as well as the barriers and enablers to successful implementation. In three specific case studies (Barcelona, Padua and Salzburg), this is done through multi-sectoral social innovation actions we refer to as ‘Resilience Hubs’. Results will be summarised in academic publications, a series of sector-specific guides, and an overall ‘What works’ guide for practitioners, policy makers, and the public.

**Discussion:**

We use a novel theoretical framework to structure a research and innovation programme to inform the implementation of nature-based therapies across Europe and globally. Challenges include the integration of terminology and research practices from multiple disciplinary perspectives, participant recruitment and attrition, especially among marginalised groups, a potential lack of local stakeholder time and interest, potentially small effect sizes of time-limited interventions, and difficulties in identifying distinct causal mechanisms. Mitigation strategies are discussed.

**Trial registration:**

Of nine case studies (CSs), five are intervention trials and have been registered:

a) CS4 doi.org/10.1186/ISRCTN74582097 (22.07.2024);

b) CS5 doi.org/10.1186/ISRCTN14169596 (10.06.2024);

c) CS6 clinicaltrials.gov/study/NCT06622629 (30.09.2024);

d) CS7 doi.org/10.1186/ISRCTN93192592 (29.05.2024);

e) CS8 clinicaltrials.gov/study/NCT06205940 (15.05.2024).

All nine case studies have received ethical approval (see Declarations section).

**Supplementary Information:**

The online version contains supplementary material available at 10.1186/s13690-026-01903-5.

**Table:** 

Text box 1. Contributions to the literature
• Despite growing interest in nature-based therapies for various health conditions there is a lack of good causal evidence of their efficacy, cost-effectiveness, or implications for sustainability/equity.
• We describe an integrated work programme designed to test a theoretical model that argues nature-based therapies work by building and maintaining biopsychosocial resilience.
• The paper provides a roadmap for how evaluations of complex public health nature-based interventions can be underpinned by theory and use multi-sectoral place-based collaborations.

## Background

### Context: nature-based solutions and therapies

Contact with nature provides a wide range of therapeutic benefits, especially for urban populations disconnected from the natural world in their daily lives [1, 2]. Interventions to increase nature contact range from substantial infrastructural changes that potentially affect entire populations such as city greening efforts [3] or increased public access to waterways [4] to programmes targeting individuals and groups with specific health challenges [5, 6]. Green/blue infrastructural projects are often referred to as ‘nature-based solutions’ [7], and they can benefit health and well-being by mitigating environmental threats such as urban heat islands, air and noise pollution, and flooding, and by helping to reduce health inequalities. By contrast, programmes targeting specific health conditions are variously referred to as ‘green care’ [8, 9], ‘blue care’ [10], ‘green prescriptions’ [11, 12], ‘green social prescribing’ [13], ‘nature prescriptions’ [5, 14, 15], ‘nature-based social prescribing’ [16–18], ‘nature-based interventions’ [19–21], or simply ‘nature-based therapies’ [22, 23]. Although various definitions exist, nature-based therapies (NbTs) can broadly be defined as planned therapeutic techniques performed in natural settings and based on nature–human active participation and connection [24]. While nature-based solutions (NbSs) can be broadly conceptualised as ‘bringing nature closer to people’, NbTs can be generally thought of as ‘bringing people closer to nature’.

Drawing on the existing evidence base, several frameworks outlining the mechanisms behind the health and wellbeing benefits of NbSs and NbTs have been proposed [25–28]. In addition to regulating ecosystem services (e.g., temperature control, [29], and mitigating harmful environmental exposures (e.g., noise, [30]), natural infrastructure and nature contact have been proposed to benefit health and well-being through several potentially co-acting mechanisms: (1) providing microbially diverse settings and stimulating Vitamin D production, both of which can support a healthy immune system [31–33]; (2) affording, and thus encouraging, health promoting behaviours such as regular time spent outdoors in various forms of physical activity [34–37]; (3) providing opportunities for relaxation and stress reduction [38, 39], which may indirectly reduce maladaptive stress reduction behaviours such as smoking [40, 41]; (4) helping to manage chronic and acute pain [42, 43]; (5) promoting better sleep [44]; (6) offering locations where people can socialise, share common activities, and build social capital [45, 46]; and (7) building place attachment and a sense of belonging [47, 48]. Common to all these mechanisms is the notion that nature supports people in building and maintaining adaptive resources that both promote and protect health and well-being in times of challenge and stress (e.g., during the COVID 19 pandemic, [49]). In other words, nature promotes resilience.

### Nature-based biopsychosocial resilience theory (NBRT)

According to nature-based biopsychosocial resilience theory (NBRT), biopsychosocial resilience, like any other resource, can be considered in terms of ‘stocks’, the underlying pool of resources at any given point in time, and ‘flows’, the processes by which resources are added to or subtracted from the stocks [50]. In terms of resilience, stocks reflect the adaptive resources (e.g., a healthy immune system, emotion regulation capacity, social bonds) present in individuals or communities that can be used in times of stress. Resilience flows reflect processes that, on the one hand, help to build and maintain these stocks (e.g., eating nutritious food, sleep and rest, physical activity, learning and experience, positive social encounters) and, on the other hand, concern their deployment before, during and after times of stress (e.g., pre-emptive actions, creative thinking, seeking social support). Unlike some stocks and flows which relate to the same underlying metric (e.g., money in economic models), the in-flows in the NBRT framework (i.e., things that support the building and maintenance of resilience stocks) can be substantively different from the out-flows (i.e., the thoughts, feelings and actions that are deployed in the face of a stressor). Stocks can be likened to more enduring trait-level resources. For instance, we might refer to an individual or community as “having resilience” in the sense that they generally have sufficient levels of adaptive resources available to cope with a range of stressors. Flows, by contrast, are reflected in actions and processes that imply changes or attempted maintenance of states. For instance, we might refer to an individual or community as “showing resilience” in the face of a given stressor.

According to NBRT, nature supports the building and maintenance of stocks relevant for two types of resilience, social-ecological resilience and biopsychosocial resilience. Social-ecological resilience stocks typically occur at the community and ecosystem level and are related to natural and social capital as a key aspect of NbSs and other blue/green infrastructure [51]. For instance, the capacity of an upstream wetland (i.e., a natural capital stock) to absorb excess water (an ecosystem service) following extreme rain makes a downstream city more resilient to climate change-related precipitation events [52]. Social-ecological resilience would be demonstrated by, for instance, a local community group successfully campaigning against building on the upstream wetland, which results in protecting the health of both people and ecosystems downstream. The existence of the community group demonstrates social capital stocks and the campaigning a flow of social capital resources. Thus, social resilience manifests itself here in the form of community involvement in governance issues in relation to an example of ecologically related resilience resources and processes. Stocks and flows of these kinds of resiliencies have been extensively described elsewhere [53–55] and, although important for several aspects of the current programme of work (e.g. see Sect. Environmental issues–Societal aspects and social innovation actions), our primary focus was on the far less researched resilience resources and processes at the level most associated with nature-based therapeutic interventions.

Specifically, biopsychosocial resilience stocks are primarily conceptualised at the level of individuals or close relationships. There are three inter-related aspects influenced by nature contact: (1) stocks that support biological resilience (e.g., a healthy immune system resulting from exposure to greater levels of microbial biodiversity); (2) stocks that support psychological resilience (e.g., enhanced emotion regulation capacities thanks to nature’s positive attention-capturing properties); and (3) stocks that support social resilience (e.g., closer partner bonds established from shared positive nature experiences; for examples of all three aspects see [50]).

**Fig. 1:**
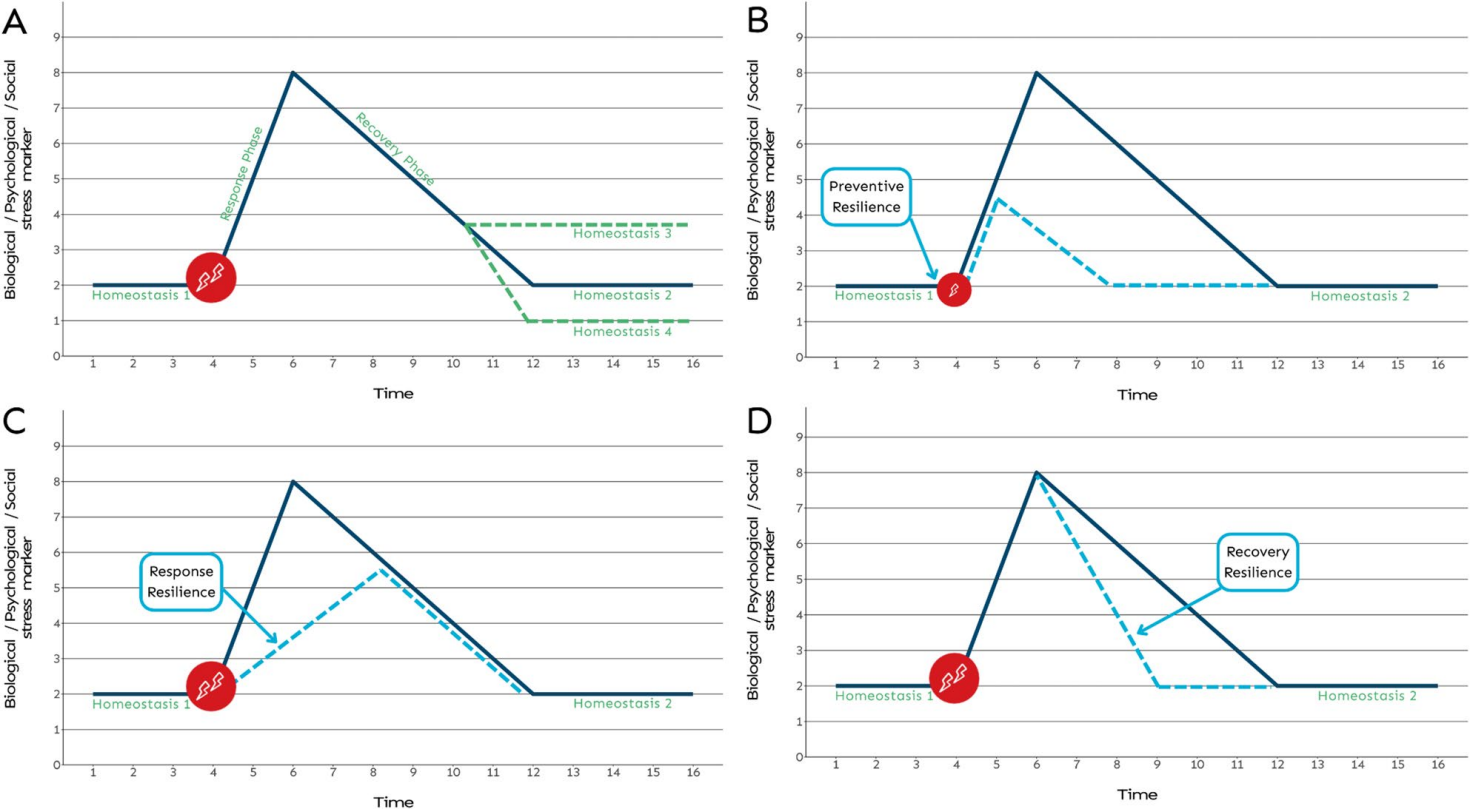
Schematic of the stress-response-recovery process and different types of resilience to mitigate total stress experienced over time. Notes: Panel (**A**) shows the stress-response-recovery cycle where homeostasis 1 is disrupted by a stressor resulting in a response phase characterized by an increase in biopsychosocial stress markers, a recovery phase, and a return to either a homeostasis similar to that before the stressor (homeostasis 2), a worse state (homeostasis 3), or better state (homeostasis 4); panel (**B**) demonstrates preventive resilience such that the potency of the stressor is mitigated resulting in a reduced response and quicker recovery to Homeostasis 2; panel (**C**) demonstrates response resilience such that the initial reaction to a stressor is muted resulting in a lower peak but similar recovery trajectory; panel (**D**) demonstrates recovery resilience such that the initial reaction to a stressor is the same but recovery is quicker. Slopes are idealized and in reality, are likely to be non-linear and different for different stress markers

These resilience stocks can be drawn upon in times of challenge or stress. Established frameworks describing resilience processes (e.g. [56]) often start at the point of a health or well-being homeostasis being disrupted by a stressor. Exposure to the stressor initiates physical, psychological and social stress reactions that persist during a response phase until the start of a recovery process. Recovery may return the person to their original homeostatic baseline, or, alternatively, may result in a worse or better level of health/well-being (Fig. 1A).

Preventive resilience refers to situations where stocks of resources are utilised (i.e., ‘flow’) to prevent or at least mitigate the scale of a stressor. In Fig. 1B, for instance, preventive resilience processes have diminished, but not eliminated, the effect of the stressor (represented by the lightning bolts), resulting in a more muted response. Many NbSs are designed with this stage of resilience in mind, for example to prevent or mitigate excess heat, flooding, or pollution affecting a specific population. Some NbTs also have preventive resilience properties. For instance, a green exercise programme may improve musculoskeletal fitness (biological resilience resources) and reduce the risk of chronic back pain [57] associated with, for instance, an otherwise sedentary job and lifestyle.

Response resilience refers to the initial reaction to a stressor occurring up to the point of a hypothetical maximal stress response. In terms of socio-ecological resilience, one example might be a community which, perhaps due to past experience, quickly and efficiently implements flood risk preparedness plans (e.g., early support for particularly vulnerable households), which lessens the impact of a flood event ([58], Fig. 1C). NbTs can also support response resilience. For instance, a recent neuroimaging study found that people reported feeling less pain following identical electric shocks when viewing a natural versus matched urban or indoor scene and that these self-reports were underpinned by lower activation in the neural circuits associated with early (pre-cognitive) pain processing [43]. The authors speculated that the natural scene supported mindful engagement with nature, an emotion regulation strategy referred to as attentional deployment [59], which reduced intensity of the pain-related neurological stress reactions to the electrical stimuli.

Finally, recovery resilience refers to the phase after maximal impact when the ecosystem, community, or individual return to a previous baseline or perhaps new homeostatic state (Fig. 1D). In terms of socio-ecological resilience, and with respect to our flood example, this might be a community returning to normal functioning quicker than a similar community due to better social capital. In terms of individual recovery resilience, perhaps the best-known example in this field is a study by Ulrich et al. [60] which showed that watching nature versus urban videos after a stressor led to a quicker and more complete recovery in terms of physiological stress markers such as galvanic skin response, heart rate, systolic blood pressure, and facial muscle tension.

In sum, NBRT details how nature, through NbSs and NbTs, can support the building and maintenance of a range of socio-ecological and biopsychosocial resilience resources, which are then drawn upon at various stages in the stress-response-recovery cycle. Although several studies have explored specific elements of nature’s resilience potential (see [50, 61] for reviews), no systematic programme of work has yet explored these processes using an integrative framework. The aim of the current paper is to outline such a programme.

### The RESONATE project

RESONATE (Building individual and community RESilience thrOugh NATurE-based therapies) is funded by the European Union’s Horizon Europe research and innovation programme, with additional funding from UK Research and Innovation. It is a 4-year (2023–2027) project including 14 partners across nine European countries (Austria, Belgium, Bulgaria, Denmark, Italy, the Netherlands, Spain, Sweden, the UK) with links to relevant initiatives in Australia, Canada, and the USA through representation on the advisory board. Building on an initial review of existing implemented peer-reviewed NbT programmes globally, a core element of RESONATE is its nine NbT case studies (CSs). While focusing on biopsychosocial resilience processes, we identify populations of interest using the health promotion/disease prevention pyramid that places populations at the bottom of the pyramid (Level-1), at-risk groups in the middle (Level-2), and those with existing conditions at the top (Level-3; [50, 62]). Accordingly, we developed three CSs for each level (nine in total). Although Level-3 CSs are the closest to notions of ‘therapy’ as a treatment, we use the term in a broader sense in that all nine CSs explore nature’s therapeutic potential.

NbTs are ‘*complex interventions*’ [63]. This makes them challenging to evaluate because they are characterised by multiple interconnected causal processes and a potentially wide range of intended (see Sect. 1.1. for NbT-relevant examples) and unintended outcomes. For instance, if people feel pressured to engage with NbTs, this can undermine intrinsic motivation and potential benefits [64], barriers to participation could exacerbate social inequalities [63], and NbTs may directly put pressure on the natural environment in which they take place. Evaluations of complex interventions are also concerned with: (1) exploring theories of how change occurs, in our case using NBRT; (2) the context in which interventions take place, including both the physical setting (here, often outdoors in natural areas), and social context (here, stakeholders include health and social care professionals, land-owners, land-managers, and the general public among others); and (3) how evidence can be translated into practice. RESONATE thus seeks to understand not just whether and how NbTs ‘work’ for individual participants but also to evaluate and address their social justice and environmental implications. In terms of our underlying theoretical model, NBRT, we thus explore not just the stocks and flows of biopsychosocial resilience for those taking part in the cohorts or interventions, but also the wider stocks and flows of social-ecological resilience in terms of broader equity, environmental, and governance issues.

### Aims and objectives

Guided by NBRT, RESONATE aims to investigate how NbTs, as complex interventions, might support the building and maintenance of not just individual biopsychosocial resilience, but also community resilience in terms of equity, social acceptability, and ecological and financial sustainability. Our interest is not just in exploring whether NbTs promote biopsychosocial resilience in the individuals engaging in them, but also in uncovering the mechanisms underlying how and why these processes work, their relative effectiveness and cost-effectiveness, and their impact beyond the individuals directly engaged (i.e., socio-ecological resilience of communities and ecosystems).

We have four broad objectives: (1) to develop a stronger evidence base regarding causal relations between nature and biopsychosocial resilience resources and processes; (2) to gain a clearer understanding of the cross-sectoral linkages spanning health, environment, economy, and wider society, required for successful NbTs; (3) to raise awareness among the wider public and sector-specific policymakers of the potential of NbTs to build and maintain a range of social-ecological and biopsychosocial resilience-related resources that can be used for preventive, response and recovery resilience; and (to the extent that high quality evidence of potential cross-sectoral acceptability and benefits emerges) (4) to support the wider utilisation of equitable, sustainable, socially acceptable, and financially viable NbTs for health promotion, disease prevention, and pro-environmental goals.

## Methods/design

Given that much of the work was still on-going at the time of submission, below we use the past tense where specific tasks have already been completed, and the present tense where tasks are still underway.

### A glossary of key terms

As noted above, a plethora of terms exist for what we refer to here as NbTs. Other relevant terminology is also diverse and used differently across disciplines. Consequently, we created an agreed-upon glossary of key terms that project partners would use to aid within-project communication and consistency in dissemination. A multi-round Delphi-style approach was used with representatives from all partners and advisory board members. Key terms relevant to the current paper can be found in the Supplementary Materials. We recognise that other definitions may exist and make no claims about our agreed terminology being pre-eminent or definitive.

### A global perspective

#### Evidence reviews

Many NbT programmes already exist, including those involving current partners and advisory board members in Australia, Canada, and the USA. To ensure this evidence base informed the proposed research, social innovation actions, and impact activities, a suite of sector-specific reviews of existing NbT programmes were conducted (see sections below), with initial searches conducted by a single team to aid standardisation following a consensus process to identify relevant terms.

In addition, we are developing an overarching ‘Systematic Map’ of existing NbTs. This focuses on *NbT programmes that have been assessed with findings published in the peer-reviewed literature* to clearly distinguish it from reviews that are looking at: (1) NbSs; (2) individual experiments rather than whole programmes; and (3) NbT programmes discussed in the grey literature. Unlike traditional systematic reviews which attempt to review the weight of evidence in support of a specific research question, this uses a systematic mapping approach [65] to scope, or ‘map’, the field in more generic terms consistent with our wider goals (i.e., not just efficacy). These dimensions include geographical location, nature-setting, target-population, stakeholder involvement, funding mechanisms, and health/well-being outcomes. This review, as well as the sector-specific ones conducted on specific themes, follow standard review protocols as set out by the Collaboration for Environmental Evidence [66] to reduce potential bias. Results of the systematic mapping review are being converted into an interactive geographical map, with results digitised in an online Geographical Information System (GIS) service to visualise NbT patterns globally. Users can zoom in and out and click on intervention icons for more details, including hyperlinks to the published papers. The map aims to serve as a repository to be used in multi-sector collaborations, for inspiration, and for guidance at local, regional, national, and international levels.

#### RESONATE global spotlight series

A preliminary version of the interactive map is being used to facilitate a series of place-specific multi-sectoral discussions of NbTs globally. This ‘Nature-based Therapy Global Spotlight Series’ aims to bring together local actors engaging in NbT research and/or practice who may or may not be aware of each other or the global picture. The series includes both in-person, online, and hybrid events in local languages; researchers, stakeholders, practitioners, policy makers, and service users are invited to discuss the use of NbTs in their region, as well as barriers and enablers, plans, and aspirations. At the time of writing, events have taken place in Italy, Singapore, the UK, Brazil/Ecuador, South Korea, Germany/Austria/Switzerland, South Africa, the USA, Canada, Malawi, and Spain with others in planning e.g. Australia. Feedback from the spotlight series will inform the final design and content of the interactive map before it is published online for full public access.

### Case studies

The nine Case Studies (CSs) are exploring how nature supports people’s ability to build and maintain biopsychosocial resilience resources that can mitigate a range of different stressors. As noted above, our aim was to have three CSs at each level of the health promotion/disease prevention pyramid. The three Level-1 general population CSs (i.e., CSs 1–3) are analysing data from two existing longitudinal cohorts and a prospective longitudinal cohort; the three Level-2 CSs (i.e., CSs 4–6) are focusing on ‘at-risk’ groups and use a matched randomised controlled trial (RCT) design; and the three Level-3 CSs (i.e., CSs 7–9) are working with individuals with existing health and well-being issues using two RCTs and a multi-site practice co-creation intervention. All CSs received ethical approval, and all RCTs were registered before data collection started (see Declaration section for details).

Three types of parameters are utilised across all CSs: (1) well-being or health-related quality of life (HRQoL) metrics; (2) measures of individual-level biological, psychological, social resilience resources; and (3) measures of community-level social and ecological resilience resources. Wherever possible, identical or similar tools are being used across CSs to facilitate statistical and narrative synthesis. For instance, we use the Short-Form Health Survey 12-Item (SF-12) to measure HRQoL in CSs 2,4,5,6,7,8, and the closest available proxy in CSs 1 (due to data availability) and 3 (due to cost), i.e., the 12-item General Health Questionnaire (GHQ12). The operationalisations of social-ecological resilience were selected collaboratively by the entire team to ensure we explore the wider range of health equity, environmental, economic, social, and process issues of interest. A CS overview, including data being collected, can be seen in Table 1. Due to space constraints, full technical details (e.g., preceding power calculations, detailed recruitment, and implementation methods) are not included here, but much of this detail can be found in the open-access trial pre-registrations (see Declarations section for links) and will be presented in detail in CS specific protocols and outputs.

**Table 1:** RESONATE Case Study 1–9 Summaries (including an indicative but not exhaustive list of measures)

Case Study	Design Prevention level (Fig. 1)	Sample*	Stressor/ risk mitigated by nature	Nature contact / NbT intervention	Health/ well-being outcomes	Biopsychosocial resilience mechanisms	Social-ecological resilience outcomes/ mechanisms
1. UK, Population representative	*Level-1*: 11 year. long. cohort	N~ 37,000	Stressful life events (income volatility)	Urban, rural, coastal neighbourhoods Occupational	GHQ-12 Life sat.	*Psych*. GSE *Social*. NSCS	*Equity.* Moderator and stratification analysis (e.g., by gender, age, etc.)
2. UK, Dementia risk Cohort	*Level-1*: 10 year. long. cohort	N~ 24,000	COVID-19	Urban, rural, coastal neighbourhoods Occupational Recreational	SF-12 GAD-7 PHQ-9	*Psych*. NAT, *Social*. Soc Support	*Equity.* Moderator and stratification analysis (e.g., by gender, age, etc.) *Envi*.^†^GEBS
3.Bulgaria, bespoke panel	*Level-1*: 12mth. long. Cohort (3 waves, 6 months apart)	*N* ~ 1,500 incl. *n* ~ 250 bio-marker cohort	Everyday stressors (e.g., social difficulties, financial, housing and environmental worries)	Urban neighbourhoods Recreational	GHQ-12 EQ-5D-5 L IPAQ-SF WHO-5 Self-rated general health	*Bio*. Immune function (e.g., IL-6), Metabolic function (e.g., lipids) *Psych*. STARS, ROS *Social*. RAS	*Envi*. INS, RPEBS *Econ*. QALY *Social*. PNSCS
4. Italy, Padua urban woods	*Level-2*: Two-arm RCT (Intervention vs. waiting control)	*N* = 134*	Having or being at-risk of metabolic syndrome (larger waistline, high blood pressure, abnormal blood lipid levels, high blood sugar	5-week guided + self-guided nature immersion Basic design in each locality will be enriched with locally supported SIA insights. CS4 Urban; CS5 Rural; CS6 Coastal Data collection: Pre (week 0), post (week 5/6), follow-up (week 10/11); CS5 only: 6-month follow-up (week 24)	SF-12 IPAQ-SF ONS-4 SPANE	*Bio*. Chronic stress (allostatic load), Immune function (e.g., IL-6/10), Metabolic function (e.g., SAA) *Psych*. STARS *Social*. BSCS, LS	*Equity*.HIAs *Envi.* EIA, INS, NCI, RPEBS *Econ*. QALY *Social.* SAS; Governance mechanisms via Resilience Hubs & process evaluations.
5. Austria, Alpine mountains	*Level-2*: Two-arm RCT (Intervention vs. waiting control)	*N* = 134*					
6. Spain, Barcelona seafront	*Level-2*: Two-arm RCT (Intervention vs. waiting control)	*N* = 134*					
7. Sweden, Urban gardens	*Level-3*: Four-arm RCT (ReST vs. mindful; vs. nature; vs. waiting control)	*N* = 260*	Clinically elevated psychological symptoms (DASS-21)	5-week ‘Restoration Skills Training’ (ReST) = formal mindfulness training + nature immersion. Data collection: Pre (week 0), post (week 5/6) follow-up (week 10/11); 6-month follow-up (week 24)	SF-12 DASS 21	*Bio.* HRV *Psych*. Attention (CFQ), STARS *Social*. LS	*Equity*. HIA *Envi*. EIA, INS, RPEBS *Econ*. QALY *Social*. SAS, Governance mechanisms via process evaluations.
8.Denmark, Urban forest/park	*Level-3*: Two-arm RCT (Visits with and without guided app)	*N* = 110*	Chronic mobility issues (e.g., wheelchair users)	5-week technology enhanced nature immersion; Week 1 in the Move Green Urban Forest; Weeks 2–5 locations near residence. Data collection: Pre (week 0), intervention (week 1; includes 3 before walk, during walk, after walk), interviews (week 2), post (week 5/6), follow-up (week 10/11), 3-month follow-up (week 17)	SF-12 SPANE	*Psych*. Attention (Eye tracking), STARS *Social*. BSCS	*Equity*. HIA *Envi*. EIA, INS, NCI *Econ*. QALY *Social.* SAS, Governance mechanisms via Process evaluations.
9.Netherlands, Care farms	*Level-3*: Standard vs. co-created Enhanced practice	*N* = 12 care farms (*N* ~ 250)	Cognitive impairment (e.g., dementia)	Co-created staff training for enhanced support of client centred nature-based experiences. Data collection: Pre (week 0), during (various), post (up to week 26).	WHO-5	*Psych*. STARS, PRS *Social*. UWES	*Equity*. HIA *Envi*. INS *Social*. CCS, PCC, SAS, governance mechanisms via process evaluations.

* Targeted N based on power analysis with expected 20–30% attrition – see specific CS protocols for more details

*Envi.*^*†*^ Environment, incl. connectedness/behaviours, *BSCS* Brief Sense of Community Scale, *CCS* Caring Climate Scale; CFQ: Cognitive Failures Questionnaaire, *DASS21* Depression, Anxiety & Stress Scale-21, *EIA* Environmental Impact Assessment, *EQ-5D-5 L* EuroQol Health Group Questionnaire, *GAD-7* Generalised Anxiety Disorder-7, *GEBS* General Ecological Behavioural Scale, *GHQ-12* General Health Qaire-12, *GSE* General Self-Efficacy Scale, *HIA* Health Impact Assessment, *IL6/10* Interleukin 6/10, *INS* Inclusion of Nature in Self, *IPAQ-SF* International Physical Activity Questionnaire Short Form, *LS* Loneliness Scale, *NAT* Network Attention Task, *NbT* Nature-based Therapy, *NSCS* Neighbourhood Social Cohesion, *ONS-4* Office of National Statistics Personal Well Being Measures – 4 item, *PCC* Person-Centered Climate Questionnaire, *PHQ-9* Patient Health Questionnaire − 9, *PNSCS* Perceived neighbourhood Social Cohesion Scale, *PRS* Perceived Restorativeness Scale, *QALY* Quality Adjusted Life Year, *RAS* Resilience Appraisals Scale, *ROS* Restorative Outcomes Scale, *RPEBS* Recurring Pro-Environmental Behaviour Scale, *SAA* Serum Amyloid A, *SF-12* Short-Form Health Survey, *SAS* Social Acceptance Survey, *SPANE* Scale of Positive/Negative Emotions, *Soc Support* Duke-UNCS Functional Social Support Questionnaire, *STARS* State-Trait Assessment of Resilience Scale, *UWES* Utrecht Work Engagement Scale, *WHO-5* World Health Organisation 5-Item Well-being Scale

#### Level-1 CSs

CSs 1–3 use longitudinal data, tracking samples of general populations over time, to monitor nature contact (e.g., neighbourhood greenness, nature visits), HRQoL, and biopsychosocial resilience before, during, and after stressful circumstances. The large samples allow us to ask research questions such as: (a) Does nature contact mitigate (buffer) the effect of stressors on health and well-being; (b) is this process mediated by biopsychosocial resilience; and (c) how similar are effects across different societal groups? Of note, CS1 and CS2 rely on secondary datasets that were designed by others before the development of NBRT and thus we selected existing variables as proxies of our constructs of interest. CS3, by contrast, has been developed within the project allowing greater freedom to operationalise some of the theory’s constructs. Statistical analysis of these CSs will also include a range of individual- and area-level variables to control for potential confounds.

In CS1, we are using four waves of longitudinal data from the UK’s Understanding Society panel survey ([67]; *N* ~ 37,000) to explore whether neighbourhood nature mitigates the negative effect of income instability on mental health, and whether this is because longer-term exposure to nature is associated with greater self-reported self-efficacy (a psychological resilience-related resource). Absolute instability in income, as well as downward volatility (decreases in income), have been observed to predict poor health and well-being [68]. We hypothesised that people living in neighbourhoods with more greenspace, living nearer to accessible greenspace, and/or living nearer to the coast (defined using land use and other spatial data), would have higher self-efficacy, which in turn would help them cope better with a change in income (manifested as a smaller drop in well-being following the income shock).

In CS2, we are using data from the first 10 years of PROTECT, a prospective longitudinal study of dementia risk in older adults living in the UK ([69]; *N* ~ 24,000), to explore whether nature contact prior to the COVID-19 pandemic mitigated its negative effects on mental health. Although numerous studies have shown that greater access to nature during the pandemic was associated with better well-being (e.g., [49]), the nature-based resilience mechanisms underlying these effects have largely focused on emotion-related processes (e.g., [70]). CS2 investigates whether more cognitive resilience processes may also play a role. For instance, drawing on attention restoration theory [71], which suggests that greater exposure to nature can help build and maintain attentional resources, we hypothesised that people with more neighbourhood nature (measured using the same metrics as CS1) would show more consistent performance on standardised attention tasks before, during, and after the peak lockdown period. Our theorising was based on the possibility that more robust attentional resources might aid processing of complex information relating to the progression of the pandemic and implementation of pandemic-related guidance, which in turn would result in more resilient mental health outcomes over time.

CS3 is a three-wave prospective longitudinal study in Plovdiv, Bulgaria, that began with a sample of *n* = 1,506 individuals. Initial power calculations suggested this would be sufficient to detect small effects (*d* = 0.1) between key variables in a structural equation model with 25 observed variables. Over a 12-month period (three waves) it explores whether multiple types of nature contact mitigate the effects of everyday stressors (e.g., housing and environmental problems, financial worries, social difficulties) on mental health, and which resilience mechanisms may underlie this. It also includes an initial sub-sample (*n* ~ 250) to investigate a rich selection of biopsychosocial resilience metrics including immune function (e.g., IL-6/10, TNF-α, CRP, TB-NK cells), stress hormones (cortisol), and general allostatic load biomarkers (e.g., cholesterol).

#### Level-2 CSs

CSs 4–6 are matched waitlist control trials targeting people either with, or at-risk of developing, metabolic syndrome, defined as a combination of at least 3 of 5 criteria (large waistline, high blood pressure, abnormal blood lipid levels, low levels of High Density Lipoprotein (HDL), cholesterol, high blood sugar) which in combination are associated with an increased risk of heart disease, stroke, and type 2 diabetes [72]. To explore generalisability across geographical, meteorological, cultural, and other contextual factors, the CSs are set in urban parks/woodlands (CS4, Padua, Italy), relatively mountainous settings (CS5, Salzburg, Austria), and urban coastal zones (CS6, Barcelona, Spain), referred to as NATURE-MET-P; NATURE-MET-S and NATURE-MET-B respectively.

Based on a meta-analysis of NbT experiments suggesting that nature-based mindfulness interventions may be particularly effective [73], the three CSs assess a five-week nature-based mindfulness intervention. Although an initial programme was collaboratively developed by the separate teams, further co-development took place with local practitioners to ensure local appropriateness. Thus, the basic foundations of the three CSs are the same, but there are also some contextual variations. Participants are randomised to the intervention or waitlist control groups (see Table 1). The evaluation will test whether individuals’ allostatic load and HRQoL indicators improve more in the intervention group than in the control group, and whether one or more of the resilience mechanisms measured (i.e., biological, psychological, social) in part account for any improvements. The intervention is an extension of existing mindfulness-in-nature practices [73] with three semi-structured sessions of approximately 60–90 min per week.

For example, in the NATURE-MET-S case, in week 1 all participants take part in three scheduled guided walk sessions (with a maximum of ten participants per group) led by a hiking guide and a mindfulness trainer, at different times of the day to suit their personal needs. In the following four weeks, at least four guided tours are offered per week, and participants can choose to join one of the guided tours or engage in their own mindfulness walks following the practices taught during week one. In total, 60 guided walks are available for the intervention group (with sub-groups running at three different times of the year), with everyone having the opportunity to attend at least three guided sessions per week. A similar number of sessions will be arranged for the wait-list control group. Attendance at all group sessions as well as additional personal walks are recorded and short surveys completed following each session.

Data in all three CSs are being collected pre (week 0), during (weeks 1–5), post (week 5/6), and delayed post (week 10/11). In NATURE-MET-S there is also the opportunity to collect data at a further delayed post (week 24). These follow-ups will allow us to explore the degree to which participants maintain higher nature contact post-intervention and longer-term effects on allostatic load and HRQoL. Based on preliminary power analysis and attrition rates from the most comparable previous study [74], each study aims to include at least *N* = 134 participants, *n* = 67 per condition; accounting for an estimated 25% pre-post attrition rate. A specific protocol paper covering these three interventions, including detailed power analyses, is currently being prepared.

#### Level-3 CSs

CSs 7–9 explore extensions to established NbT programmes for individuals with existing conditions or symptoms.

CS7 will build on the Restoration Skills Training (ReST) programme [75] which integrates mindfulness training with restorative nature experiences for students with symptoms of poor mental health. A four-arm RCT design will explore resilience processes by comparing ReST and a non-mindfulness-based nature-on-prescription intervention with each other and with conventional (indoor) mindfulness training and a waitlist control. An initial power analysis based on earlier comparisons between a) ReST and conventional mindfulness training [75], and b) ReST and a Green prescription intervention suggested a required minimal sample size of *N* = 260, *n* = 65 per condition, accounting for an estimated 20% pre-post attrition rate. A random subset of 100 participants is also being asked to complete a stress management test before and after the interventions to explore cognitive and psychophysiological resilience effects.

CS8 builds on the Move Green project, an initiative aimed at designing accessible forest trails with health-supporting nature experiences and developing nature-based activities for all, including individuals with mobility issues. Given that these individuals tend to visit nature less due to accessibility issues [76] and are also often more exposed to stress and have significantly poorer HRQoL, compared to people without such challenges [77], improved access to nature may help them build resilience resources to cope with these stressors. Move Green has developed a mobile phone app, and complementary web-based version, that contains guided restorative nature experiences aimed at enhancing sensory experiences, physical interactions, and connectedness with nature. The trial uses a two-arm RCT five-week intervention with two distinct phases. In week one, both the intervention and control groups visit an arboretum and are asked to follow an access-friendly trail, stopping at three carefully selected locations to sit and appreciate the surroundings. The key difference is that the intervention group receive app-based auditory information at these stopping points aimed at enhancing their experiences (e.g., through site-specific guidance and stories), while the active control group are only told to sit quietly for 5 min before moving on. In weeks two to five, participants in both arms are encouraged to continue engaging in nature visits on their own at local or preferred locations with only the intervention group continuing to have access to further engagement enhancing audio instructions. A subset wears eye-tracking devices during the Arboretum visit to improve understanding of how people with mobility issues navigate natural spaces. A power analysis suggested a minimum sample of *N* = 110, *n* = 55 per condition, accounting for an estimated pre-post attrition rate of 30%. A full protocol paper for this CS containing more technical details has been submitted for publication.

CS9 uses a co-creation approach to explore how to strengthen biopsychosocial resilience by adapting existing practices at care farms in the Netherlands that offer daycare for older adults with dementia and children with cognitive impairments. Twelve care farms are involved: six intervention sites and six controls. The intervention consists of a series of three meetings over a period of half a year with staff members who, during the meetings and in-between, share, co-design, and test innovative activities and interventions in the outdoor areas, inspired by input from experts and researchers. These activities go beyond their traditional walking and gardening routines and are tailored to the biopsychosocial resilience needs of clients. Reflecting the mindfulness elements present in other case studies, caregivers are trained to observe how clients are engaged and guided by affordances in the natural environment, as described, for example, in the model of Perceived Sensory Dimensions (e.g., [78]). In addition to mainly qualitative evidence from videos, interviews, and focus groups gathered during the co-creation process, quantitative data and observations are collected through pre–post surveys and observations among the entire staff at all farms to explore changes in client and staff well-being at both intervention and control sites. A focus on biopsychosocial resilience processes is central to both the qualitative and quantitative data collection. Although the control farms do not receive the intervention, the results will be shared with them and other farms so that they can also consider adopting practices identified by the intervention farms where appropriate.

### Core themes

As noted above, RESONATE is not only interested in participant-related outcomes but also broader issues such as outcome equity, environmental impacts (positive and negative), financial issues and social awareness and acceptability among various stakeholders including members of the public, health care professionals and those who may prescribe or fund NbTs, as well as the owners and managers of land where NbTs may take place. In addition to the required data being collected by the case studies, these themes are also being explored through a series of interviews, workshops, and surveys with relevant actors. The learnings from these different activities will be written up in a series of ‘NbT Guides’ targeting different stakeholder audiences as well as an overarching ‘What Works’ guide that brings all the themes together. This overarching guide will both draw on key information from the theme-specific guides and also include results of a detailed process evaluation of the controlled trial CSs and future scenario analyses of Level-2 CSs aimed at ‘future proofing’ potential programmes for potential social, economic, and environmental change.

#### Health and health equity

A core part of the project focuses on understanding the concerns, challenges, barriers, needs, and opportunities of the health sector related to implementing NbTs and on providing tools and guidance for health professionals. In particular, we are focusing on: (1) the health sector’s role in supporting or restricting uptake of NbTs; and (2) how health determinants and access to green/blue spaces and NbTs are distributed across the population (i.e., does everyone benefit equally? ). We began by reviewing the grey literature on NbTs and health equity (to reduce overlap with the global mapping review, see Sect. Evidence Reviews) and are conducting a series of in-depth interviews with health professionals about their awareness and perceptions of the appropriateness, acceptability, and equity of NbTs. Interviewees to date span primary care (e.g., General Practitioners), specialists/doctors in secondary care, health service administrators, and funding priority setters. They are from a range of geographies/countries (including outside the EU to learn about other systems) and have varying levels of NbT experience, including no prior experience. These processes are supported by two of the project’s advisory board members, both of whom are practicing family doctors.

We are also conducting either comprehensive or rapid health impact assessments (HIAs) for the Level-2 and Level-3 CSs depending on data availability. Data being collected include social and environmental determinants of health, and focus on site factors (e.g., air quality, noise, walkability), participant characteristics (e.g., education, employment status), and local contexts (e.g., current NbT attitudes/practices). We are following the five key stages laid out by the WHO [79] and the EU’s Joint Action Health Equity Europe [80] for health impact assessment tools: *Screening* (deciding on comprehensive vs. rapid HIAs); *Scoping* (establishing how to conduct the HIAs); *Appraisal* (supporting CSs to gather and analyse relevant data); *Reporting* (presenting results and providing recommendations); and *Monitoring* (identifying goals for monitoring and evaluating the effectiveness of the HIA process). Results of the review, interviews, and HIAs will be synthesised into a ‘*Nature-based Therapy Guide for Health Professionals*’ targeting those who are in a position to prescribe NbTs.

#### Environmental issues

Human and environmental health are deeply interconnected [81], and environmental quality is a key determinant of nature’s potential to contribute to human health and well-being. NbTs could, if not carefully managed, generate pressures on the environment itself, reducing the quality of the NbT experience with negative effects on both biopsychosocial and social-ecological resilience. Work on this topic began with a literature review of existing NbTs, using papers funnelled from the overarching search strategy (see Sect. Evidence Reviews), this time to find evidence and/or discussion of possible environmental impacts, whether positive or negative. The review informed: (1) variables that CSs were asked to collect pre, during, and post intervention; and (2) a Delphi process to develop CS specific environmental risk assessments (ERA) conducted in collaboration with local environmental experts due to the highly location specific issues of environmental impact. Based on the type of NbT intervention, biodiversity/ecosystem indicators at risk and other factors have been identified. Although we might expect relatively little direct environmental impact of trials of this nature and timescale, the ERA results are being related to the environmental elements present in each CS, to identify those characteristics and quality levels that could condition the success of NbTs, as well as levels of impact that would hinder ecological sustainability.

A key aim of this process is to make recommendations about how to adapt or modify NbT design and/or propose mitigation measures to minimise any negative environmental impacts. NbTs may also have environmentally positive outcomes. For instance, spending time in, and being mindful of, nature is associated with better nature connectedness and more pro-environmental behaviours [82, 83], and so relevant data is also being examined across multiple CSs. This includes pro-environmental attitudes and behaviours (pre, during, and post intervention) as well as potentially important individual differences such as childhood contact with nature, environmental knowledge, and nature connectedness [84]. The aim is to see how NBTs could be modified to promote environmental benefits, not just reduce environmental harms. Processes and results will be summarised in a ‘*Nature-based Therapy Environmental Assessment and Impact Guide*’ targeting those in the environmental sector concerned about or trying to understand the opportunities of NbTs for the environment, as well as ways to make NbTs environmentally sustainable from the design phase to implementation.

#### Economic and financial issues

The benefits of nature to people’s health can also be conceptualised in economic terms, by estimating, for instance, health utility valuations or avoided health care costs [85–87], which is potentially important for decision makers seeking to prioritise limited budgets. Despite demonstrable financial benefits, many NbT providers are concerned about a lack of sustainable financing [88], partly due to a lack of clear business cases available for public or private funders/investors.

We began consideration of economic issues with a review of the economically relevant literature (again using papers funnelled from the overarching search strategy, Sect. Evidence Reviews) to help identify at the CS level: (1) the asset being assessed (e.g., single ecosystem service, co-benefits, nature in general); (2) the reference population (who benefits); (3) the most appropriate/feasible evaluation method (e.g., cost-based, demand-based, cost-effectiveness and/or cost-benefit methodologies); (4) the sampling plan; and (5) definitions related to economic impact. Analysis will focus on the extent to which CSs might save health and social-related costs for their users, society, and public/private health institutions. For CSs with clear current practice comparators (e.g., ReST vs. conventional mindfulness training in CS7), cost-effectiveness analysis will also be undertaken.

Although the economic potential of any single trial is likely to be small in absolute terms, the synthesised data will be used to develop evidence-based scenarios about what scaled-up or scaled-out offerings of selected NbTs might look like. Based on the CS results, it will be possible to quantify the hypothetical impact of a generalised adoption of NbTs, for example considering job creation and local economy improvements, and to identify, catalogue, and assess business cases and the market for NbTs. Such results are important for demonstrating the value of NbTs. Processes and results will be summarised in a ‘*Nature-based Therapy Economic Impact Assessment Guide*’, targeting those in, for instance, the health field who want to understand the relative cost-effectiveness of NbTs compared to other possible programmes they could prescribe to their patients.

We are also collating and synthesising potential sustainable financing options. Developing the public and private business and market case for NbTs requires a market analysis of the supply and demand potential for NbTs in different sectors (e.g., green space management, agriculture, forestry) and in the public/private sectors, including health insurers. Such an analysis includes the barriers and opportunities for accessing public/private finance for NbTs and what makes them bankable and will result in a ‘*Nature-based Therapy Sustainable Financing Guide*’, targeting those potentially interested in investing in NbT programmes.

#### Societal aspects and social innovation actions

NbTs often take place in publicly accessible/shared spaces, and thus other users (e.g., recreational visitors) are also stakeholders. Understanding their perspectives, as well as those of the local community more broadly, is critical for the long-term acceptability of NbTs. Regardless of type, successful NbTs tend to have a core social element, including community engagement in green/blue-infrastructural design [89], and group-based nature activities [13]. Consequently, some NbTs can be conceptualised not just as complex interventions but also as ‘social innovations’ [90]. Although the term has a multi-disciplinary heritage, social innovations tend to reflect social processes that are built on the voluntary engagement and collaboration of citizens to help create new social networks, civil society partnerships, and social entrepreneurships that help deliver services to vulnerable groups (e.g., [91]).

In this context, we aim to contribute to a better understanding of factors affecting awareness and societal acceptability of NbTs and facilitating the establishment, implementation, and scaling of selected NbT interventions through a series of social innovation actions (SIAs). We meet this aim by: (1) identifying and examining the factors that promote/hinder awareness and social acceptance of NbT interventions in different socio-economic, institutional and geographical contexts; (2) identifying linkages and gaps between healthcare, social, and educational sectors and green space management, nature protection, agriculture, and forestry sectors for the contexts of the three Level-2 CSs (i.e., 4–6); and (3) facilitating the design, implementation, and or scaling of initiatives linked to these CSs to help local communities turn NbTs into opportunities for community resilience, green job creation, and nature protection.

Due to the centrality of both biopsychosocial and social-ecological resilience in our approach we refer to these SIAs as NbT ‘Resilience Hubs’. The three place-based demonstrator Resilience Hubs, one associated with each of the Level-2 CSs (i.e., CSs 4–6), have built on, strengthened, and enlarged existing local networks to establish and run local NbT initiatives by empowering individuals and existing groups to be partners in the NbT innovation processes. Drawing on best-practice guidance from the Social Innovation in Marginalised Rural Areas (SIMRA) project [92], stakeholders include actors from all relevant sectors, for instance, health/social care, planning, agriculture, forestry, marine, education, public/private finance, policy makers and resident associations. In each Resilience Hub, a preparatory social network analysis was conducted using snowball sampling techniques, questionnaires, semi-structured interviews, and specialised software, to analyse the existing cross-sectoral linkages and identify the potential gaps in relations between key actors. The Resilience Hubs have since been acting as focal nodes for community engagement, guiding the cross-sectoral co-creation process, and seeking to find new governance solutions, new shared values and attitudes, and financial instruments to support a stable reconfiguration of nature-based social practices for more locally resilient communities.

Combined, these activities aim to support our understanding of how NbTs can be scaled-up (e.g., changing policies/regulations to support NbTs) and scaled-out (e.g., increasing numbers, rolling out to different locations and other vulnerable groups). Lessons learned from the three demonstrator Resilience Hubs will be synthesised in a ‘*How to Set up Resilience Hubs for Nature-based Therapies Guide*’, targeting local actors interested in moving beyond fragmented and/or small-scale programmes that focus primarily on the health, well-being, and resilience of a small set of individuals, to larger, more coherent programmes also concerned with community-level resilience and well-being.

### Bringing it all together: what works - a cross-sectoral view of effective, equitable, replicable, sustainable & scalable NbTs

In order to clarify the factors that contribute to whether or not NbTs ‘work’ in the broadest possible sense and using empirical data to inform our NBRT model, RESONATE will also synthesise the various elements, including the systematic reviews, the results of the CSs, the outcomes from the thematic specific interviews, workshops, and surveys, and the specific guides. There are four core elements to this integration:

#### Quantitative integration via multi-study meta-analyses

Due to our coordinated data collection protocols, multiple studies use the same measures pre-post so that their data can later be synthesized. This data will be used in several multi-case study meta-analyses to explore the effects of condition (intervention vs. control) on different aspects of both (1) individual level resilience markers (including biological, psychological and social parameters) and (2) broader social-ecological resilience metrics (e.g. equity, ecological risk, cost-savings). Adopting a coordinated analytical approach, including Linear Mixed Effect Models (LMMs) and structural equation models (SEMs), different teams will lead on meta-analyses of specific relevance based on areas of expertise (e.g. biological markers) or themes (e.g. pro-environmental behaviours).

#### Qualitative integration via the process evaluation methodology

To help the process of integrating the various learnings across the different themes, we developed a systematic process evaluation protocol [93] for Level-2 and Level-3 CSs that will examine: (1) the implementation of each CS (i.e. why participants enrol or drop out, the routes and appropriateness of referrals, and the extent of participation/engagement with the interventions); (2) the context of each CS (i.e. the individual-level, intervention-level, and contextual factors that affect delivery, uptake, and participation); and (3) the mechanisms of impact (i.e. the biopsychosocial processes that may have brought about a change in the primary outcomes).

Data collection and analysis will primarily be qualitative, utilising participant and practitioner interview data. For CSs 4–7 we aimed to conduct interviews with *n* ~ 10 participants from each intervention arm (total *n* ~ 40) and 2–3 intervention deliverers (total *n* ~ 10). For CS8, short qualitative interviews will be conducted with each participant following the initial guided walk in Week 1, and open-ended questions included in the Week 5 (Post) survey. For CS9, qualitative work is a more integral part of the process in all farm locations and includes conversations with clients, farm support staff, and researchers in group as well as 1-2-1 settings, with the exact number varying by location. In addition, for WP4 we are conducting *n* ~ 10–15 interviews with health care professionals to discuss their attitudes towards NBTs. For workshops, e.g. Resilience Hubs, we aimed to recruit around 15–20 people per session (WP7).

The process evaluations based on these data are co-designed, e.g., in the Resilience Hubs, data collection is supported by the CS leads, and a synthesis across CSs will be produced. Findings will inform a ‘*Nature-based Therapy Process Evaluation Guide’* targeted at those trying to optimise cross-sectoral practices in future NbT programmes.

#### Quantitative and qualitative integration for future scenarios

It is also important to understand not just ‘what works’ now but how this might change in the future. Accordingly, we are also conducting cross-sectoral scenario analyses for Level-2 CSs in order to better understand how to ‘futureproof’ NbTs given potential socio-demographic and environmental changes. Scenarios are estimating the cross-sectoral impact of scaling-up and/or scaling-out a given programme under different future conditions, specifically, selected Shared Socioeconomic Pathways (SSPs) as defined in the IPCC Sixth Assessment Report [94] using fuzzy cognitive maps [95] built on information gathered during expert and stakeholder workshops. In addition, for CS6 (Barcelona), the social-ecological system of the city is being simulated using quantitative modelling techniques, to test the resilience of the system, simulate its behaviour under different future conditions, and compare management alternatives.

#### Narrative integration via the guides

Finally, a combined narrative integration of all quantitative and qualitative aspects is the task of the various Guides, of which there are two levels. At the first, sector specific, level, the guides will narratively synthesize both the quantitative and qualitative findings relevant to their thematic area. The Health Practitioners Guide, for instance, will include both high-level findings from the meta-analyses of health and well-being outcomes, and key findings from interviews with current NbT prescribers in Europe and Canada. In turn, the task of the overall “What Works Guide” will be to narratively synthesise the main findings of each of the sector specific guides and draw together key synergies and trade-offs.

### Approach and structure of the RESONATE project

**Fig. 2:**
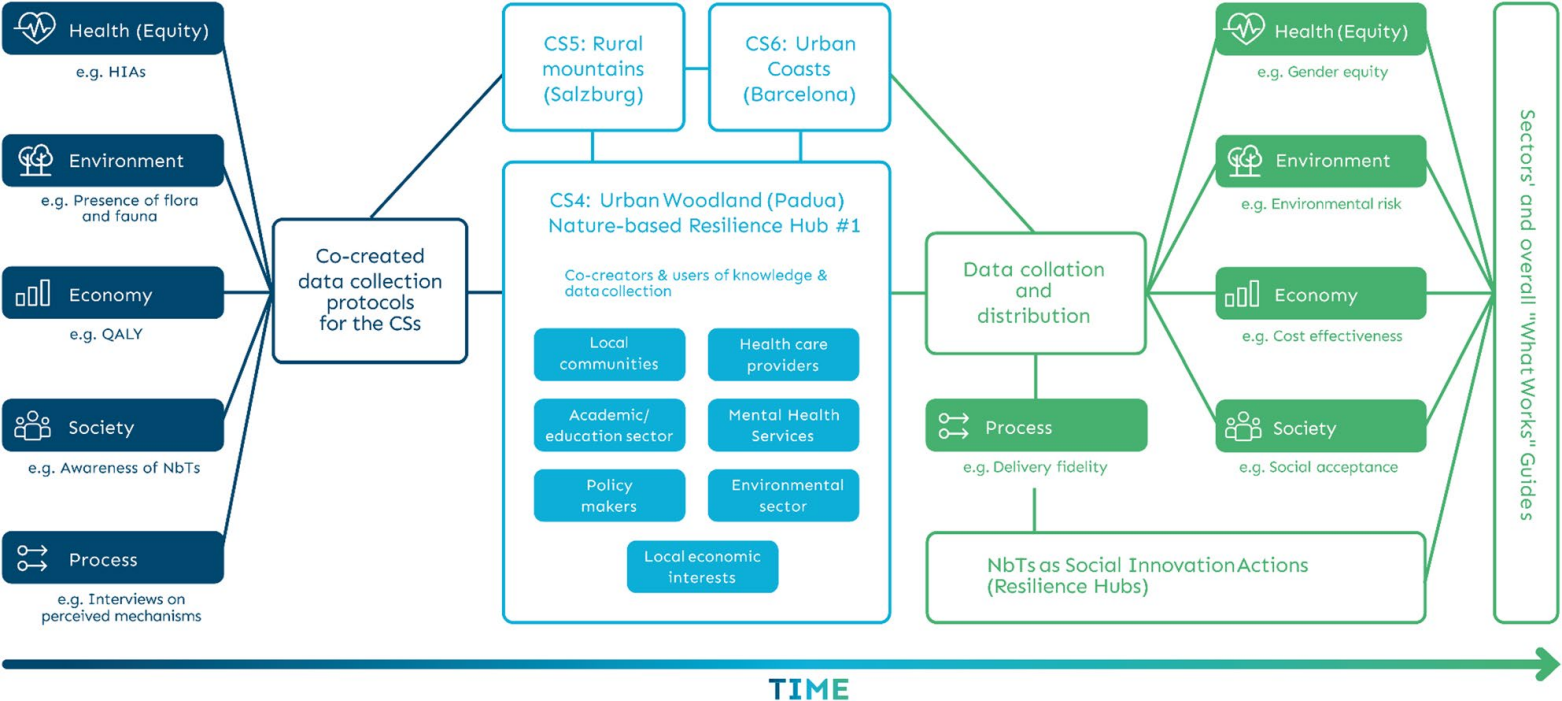
A schematic overview of cross-sectoral collaboration and research using Case Study 4 as an example. *Notes*: CS = case study; HIA = Health Impact Assessment; QALY = Quality Adjusted Life Years; NbTs = Nature-based Therapies

A schematic view of our integrated approach is shown in Fig. 2, taking CS4 as an example. The Resilience Hub is the central focal point, with members drawn from different local sectors. Once developed within the team, Resilience Hub participants were presented with the same basic NbT study design, and members asked to discuss the pros/cons and enablers/barriers of such a study in their locality. Simultaneously, other members of the consortium were developing a data needs package spanning the research objectives including those related to the themes of health, health equity, environment, economy, society, and process. A single data management system was established at the lead institution such that all CS4 data (as well as that from the other Level-2 CSs) is saved on a single secure server. Once CSs have finished data collection, the relevant data will be made available to the relevant sub-teams for analysis using secure protocols. The future scenario team will also use this information, alongside future socio-demographic and climate predictions, to develop locally relevant NbT scenarios. As noted above, sector-specific guides will be produced for different target audiences, and an overall synthesis of these guides will be produced, i.e. the ‘What Works’ guide, which will also include results of the process evaluation and future scenarios work.

The project was divided into four stages of approximately a year each; *Stage 1*: Planning and preparation, included conducting the literature reviews, local stakeholder network mapping, creation of the Resilience Hubs, adaptation of the planned interventions with local stakeholders, co-design of surveys and semi-structured interviews, and ethics applications; *Stage 2*: Data collection for CSs 3–9, and several thematic sectors, continued engagement with the Resilience Hubs, data analysis for secondary data CSs 1 and 2; *Stage 3*: Completion of data collection and data analysis, both at the CS and the thematic levels, requiring the synthesis of data across multiple case studies, and running of the Global Spotlight series; *Stage 4*: Dissemination and Exploitation, production of main deliverables (i.e. the sector specific and overarching “What works” guides), summaries of these in the form of Fact Sheets and Policy Briefs, preparation and submission of academic papers, presentation of findings at academic conferences, local feedback meetings (e.g. Resilience Hub sessions), planning and delivery of policy maker meetings.

## Discussion

Successful implementation of public health interventions depends both on a sound theoretical foundation [96] and a deep appreciation of practical implications and challenges [63]. To understand how NbTs could be optimally designed and implemented, we developed NBRT [50] and viewed NbTs as ‘complex interventions’ with multiple causal processes, a range of intended and unintended consequences, and various contextual factors that would need to be accounted for [63]. Building on these starting points, we developed a coherent programme narrative that focuses on a common set of resilience-related resources and processes across multiple case studies, social innovation actions, and areas of thematic interest to better understand how NbTs can be effective not just for those who take part in them, but also equitable, environmentally sustainable, financially viable, and actively supported by local stakeholders.

Although we believe RESONATE could produce many positive outcomes, we also recognise multiple challenges and have identified a range of risk mitigation strategies. Challenges include those associated with: (1) making coherent sense of findings from such a broad range of studies and tasks; (2) linguistic and cultural issues associated with multi-disciplinary/multi-sector/multi-country collaborations; (3) participant recruitment and attrition, including equitable participant engagement; (4) a lack of local stakeholder interest and/or limited time for engagement and co-creation; (5) potentially small effect sizes for relatively short interventions; and (6) the challenge of clearly identifying distinct causal mechanisms; which, combined, may lead to (7) resistance among different sectors (e.g., medical, land-owners) to consider the potential of NBTs further in their sector. We also recognise that this is a largely European-focused project developed by predominantly European-heritage researchers and practitioners, and we cannot assume that findings will necessarily generalise to non-Western communities within and outside Europe.

These risks have been mitigated through various mechanisms. Using a single theory to guide the creation of all elements in the programme has aided coherence at all stages of programme development and will continue to aid the synthesis of findings. Interdisciplinary respect and openness to cooperation are lynchpins in breaking down linguistic and cultural barriers, and initiatives such as the mutually produced glossary exemplify how we are reducing risk by listening and coordinating across sectors, disciplines, and academic cultures. Plans to reconsider exclusion criteria (e.g., age, number of at-risk symptoms) for CSs have been drawn up in the case of recruitment difficulties, and several strategies to recruit participants from marginalised groups have been identified [97]. The Resilience Hubs, for example, are key in this process, because by using evidence-based social innovation practices we aim to build local ‘ownership’ of the interventions and reduce the risk of recruitment challenges through engagement of a range of local stakeholders with more direct access to various target groups. In terms of analytical (statistical) power, all Level-1 CSs use large longitudinal samples and should be able to detect even small effects. In addition to power analyses, the individual RCTs (especially CSs 4–6) are designed to allow for the conduct of individual-participant data meta-analyses across the CSs that will significantly increase analytical power and enable additional insights [98, 99]. By measuring biological, psychological and social resilience metrics across multiple CSs, there is potential to tease apart the underlying causal mechanisms of any health and well-being benefits of the interventions. By measuring a range of socio-ecological resilience parameters, it will also be possible to examine the wider factors that hinder or enable success of such complex interventions. Finally, the Global Spotlight series is one way in which we address the issue of generalisability and relevance beyond Europe and learn more about global practices.

The concept of resilience lies at the heart of the RESONATE project. Our aim is to explore how nature in general, and nature-based therapies in particular, can build and maintain not only biological, psychological, and social resilience for those directly in contact with nature, but also social-ecological resilience for the wider communities in which they live. By investigating the evidence for, and barriers and enablers of, the mainstreaming of equitable, socially acceptable, and environmentally and financially sustainable NbTs, RESONATE thus aims to contribute to the building and maintenance of both individual-and community-level resilience and the promotion of wider human and planetary health.

## Supplementary Information

Below is the link to the electronic supplementary material.

Supplementary Material 1.

Supplementary Material 2.

## Data Availability

No data are presented in this programme protocol article. Except for CSs 1 and 2 which employ secondary data analysis, data collection is underway across the remaining CSs and WPs at the time of original submission. The data for CS1, the Understanding Society panel data, can be accessed here (following registration): [https://www.understandingsociety.ac.uk/documentation] . Access to the data for CS2, the PROTECT panel data, can be granted via the PROTECT team ( [support.protect@exeter.ac.uk] ). RESONATE aims to make as much of the remaining data collected publicly available after the end of the project, while taking care to maintain anonymity of participants (e.g., anonymised survey data will be made available, but interview transcripts which are harder to fully anonymise will generally not be made available). Data sharing statements will be completed in all CS and WP paper submissions where appropriate. Data is being managed using the REDCap system (https://www.project-redcap.org/), hosted on a secure server at the lead partner University. All data (except for secondary data CSs 1 and 2) is either being collected directly through the system (e.g. CSs 4-6) or being uploaded to it post collection, including qualitative interviews once translated into English. This provides a central data hub both for the researchers in the project itself (who require data from multiple CSs and WPs) and for external researcher data requests in the short-term. In the longer-term, after the project has finished, data will be uploaded to the EU’s Zenodo repository ( [https://zenodo.org/] ) and continue to be managed by the team for at least 10 years after the end of the project (i.e. June 2037).

## Abbreviations

CS: Case study
HIA: Health impact assessments
HRQoL: Health-related quality of life
NbS: Nature-based solution
NbT: Nature-based therapy
NBRT: Nature-based biopsychosocial resilience theory
RCT: Randomised Controlled Trials
RESONATE: Building individual and societal RESilience thrOugh NATurE: based therapies
SIA: Social Innovation Action
WHO: World Health Organisation
