# Supplementary material for: Testing nature-based biopsychosocial resilience theory: a research programme protocol
Source: Arch Public Health. 2026 Apr 24;84:90. doi: 10.1186/s13690-026-01903-5 (PMC13107860; doi:10.1186/s13690-026-01903-5)
Supplement: Supplementary file 1 — Supplementary Material 1. [file 13690_2026_1903_MOESM1_ESM.docx]

**Testing nature-based biopsychosocial resilience theory: A research programme protocol**

Mathew P. White*, Julia A. M. Egger, Giulia Amato, Thomas Astell-Burt, Stine Bekke-Hansen, Angel Borja, Angel Burov, Svea Busse, Caroline Costongs, Donka Dimitrova, Ilaria Doimo, Angel M. Dzhambov, Lewis R. Elliott, Alba Godfrey, James Grellier, Terry Hartig, Arnulf Hartl, Patrik Karlsson Nyed, Cecil Konijnendijk, Melissa Lem, Jill S. Litt, Rebecca Lovell, Freddie Lymeus, Aynur Mammadova, Leanne Martin, Angelica Moè, Sarah Morgan Trimmer, Colm O’Driscoll, Johan Östberg, Sabine Pahl, Christina Pichler, Francesca Pazzaglia, Alexandria Poole, Sarai Pouso, Todora Rogelja, Laura Secco, Ulrika K. Stigsdotter, Sus Sola Corazon, Georgina Sowman, Maria C. Uyarra, Agnes E. van den Berg^,^ Thomas van Rompay, Martin Voracek, Nancy M. Wells, Benedict W. Wheeler, Matilda van den Bosch

* **Corresponding author:** Mathew P. White, Institute for Clinical and Health Psychology, Kolingasse 14-16, University of Vienna, Vienna, A-1090, Austria; email: [mathew.white@univie.ac.at](mailto:%20mathew.white@univie.ac.at)

**Supplementary Material**

This file includes a glossary of terms, supporting the main article. The consortium members intend them to be interpreted in the context of the research programme.

**Table of Contents**

Allostasis 1

Allostatic load 1

Blue space 1

Blue infrastructure 1

Community 1

Ecosystem 2

Ecosystem Services 2

Equality 2

Equity 2

Forest 2

Urban forest 2

Green space 3

Urban green space 3

Green infrastructure 3

Health 3

Homeostasis 3

Nature 4

Natural elements 4

Natural setting 4

Nature contact 4

Nature-based therapy 4

Nature-based social prescribing 4

Nature-based solutions 4

Nature’s contribution to people 5

Resilience 5

Biological resilience 5

Biopsychosocial resilience 5

Community resilience 5

Preventive resilience 6

Psychological resilience 6

Recovery resilience 6

Social resilience 6

Social-ecological resilience 6

Resilience hubs 6

Social-ecological system 7

Social innovation 7

Social innovation action 7

Stakeholders 7

Stress 8

Stress response 8

Stressor 8

Social network analysis 8

Social acceptability 8

Social awareness 9

Quality of Life 9

Well-being 9

References 10-12

**Allostasis**

“maintaining stability, or homeostasis, through change” (Sterling and Eyer 1988).

**Allostatic load**

“the [bodily] cost of chronic exposure to fluctuating or heightened neural or neuroendocrine response resulting from repeated or chronic environmental challenges that an individual reacts to as being particularly stressful” (McEwen & Stellar 1993).

**Blue space**

“outdoor environments - either natural or manmade - that prominently feature water” (Grellier et al. 2017) (e.g. rivers, lakes, ponds, canals, fountains, coastal margins).

→ see also Green space

**Blue infrastructure**

“Blue infrastructure includes bodies of water, watercourses, ponds, lakes and storm drainage, that provide ecological and hydrological functions including evaporation, transpiration, drainage, infiltration and temporarily storage of runoff and discharge.” (IPCC 2022)

→ see also Green infrastructure

**Community**

A group of people who are linked by social ties, share common interests, values, and engage in social interactions to fulfil mutual needs and aspirations in geographical locations or settings (based on MacQueen et al., 2001). Together with this territorial and geographical notion of community (neighbourhood, town, city), the relational notion of community (quality of human relationships, without reference to location) is also relevant (e.g. professional links, spiritual values, etc.) (McMillan and Chavis, 1986). These notions do not exclude each other. Communities can be based on various factors, including locality (geographical communities), identity (cultural, ethnic, or religious communities), interest (professional, academic, value-guided or hobby-based communities) or virtual space (digital communities). Key elements of a community include: commonality (members share common interests, values, goals or characteristics); interaction (members engage in social interactions and relationships); mutual support (members can receive support and resources from the network they belong to), identity (members have an individual sense of belonging to the community) and shared space (this can be a physical location or virtual space).

→ see also Community resilience

**Ecosystem**

“A functional unit consisting of living organisms, their non-living environment and the interactions within and between them. The components included in a given ecosystem and its spatial boundaries depend on the purpose for which the ecosystem is defined: in some cases, they are relatively sharp, while in others they are diffuse. Ecosystem boundaries can change over time. Ecosystems are nested within other ecosystems, and their scale can range from very small to the entire biosphere. In the current era, most ecosystems either contain people as key organisms or are influenced by the effects of human activities in their environment.” (IPCC 2022)

**Ecosystem Services**

“the range of benefits provided to humans by healthy ecosystems. Services include provisioning (such as food and wood), regulating (for example climate, flood and water regulation) and cultural services (for example, spiritual, recreation, educational)” (MA 2005).

→ see also Nature’s contribution to people

**Equality**

“A principle that ascribes equal worth to all human beings, including equal opportunities, rights and obligations, irrespective of origins.” (IPCC 2022)

**Equity**

“The principle of being fair and impartial, and a basis for understanding how the impacts and responses to threats and opportunities, including costs and benefits, are distributed in and by society in more or less equal ways. Often aligned with ideas of equality, fairness and justice and applied with respect to equity in the responsibility for, and distribution of, policies across society, generations and gender, and in the sense of who participates and controls the processes of decision-making.” (IPCC 2022)

**Forest**

“Land spanning more than 0.5 hectares with trees higher than 5 metres and a canopy cover of more than 10 percent, or trees able to reach these thresholds in situ. It does not include land that is predominantly under agricultural or urban land use.” (FAO 2020) Forests include both natural forests and planted forests. It also includes areas temporarily unstocked, e.g., after disturbance, that are expected to revert back to forest (IUFRO 2023).

**Urban forest**

“The sum of all woody and associated vegetation in an urban area.” (Miller, 1988)

→ see also Forest

NOTE: This definition contradicts the general definition of Forests, which we acknowledge but accept since Urban forest is a standing term with an academic tradition.

**Green space**

Outdoor environments - either natural or man- made - that prominently feature vegetation.

→ see also Blue space

NOTE: Own definition analogous to the definition of blue space by Grellier et al. 2017.

**Urban green space**

A green space in an urban setting, such as a plot of vegetated land separating or surrounding areas of intensive residential, commercial or industrial use (adapted from EEA).

→ see also Urban forest

**Green infrastructure**

“The strategically planned interconnected set of natural and constructed ecological systems, green spaces and other landscape features that can provide functions and services including air and water purification, temperature management, floodwater management and coastal defence often with co-benefits for human and ecological well-being. Green infrastructure includes planted and remnant native vegetation, soils, wetlands, parks and green open spaces, as well as building and street-level design interventions that incorporate vegetation.” (IPCC 2022)

→ see also Blue infrastructure

**Health**

“a state of complete physical, mental and social well-being and not merely the absence of disease or infirmity” (WHO 1948)

One of the mechanisms to achieve health is

→ Resilience.

**Homeostasis**

“a self-regulating process by which biological systems maintain stability while adjusting to changing external conditions. This concept explains how an organism can maintain more or less constant internal conditions that allow it to adapt and to survive in the face of a changing [...] external environment.” (McEwen 1998)

**Nature**

Nature “refers to the natural world with an emphasis on its living components. Within the context of western science, it includes categories such as biodiversity, ecosystems (both structure and functioning), evolution, the biosphere; humankind’s shared evolutionary heritage, and biocultural diversity. Within the context of other knowledge systems, it includes categories such as Mother Earth and systems of life, and it is often viewed as inextricably linked to humans, not as a separate entity.” (Diaz et al. 2015)

**Natural elements**

Individual building blocks and relations between them that build up the natural setting and eventually the ecosystem, e.g. geological features, plants, birdsong (adapted from White et al. 2023).

**Natural setting**

Geographically contained setting within an outdoor ecosystem, e.g. park, beach, garden or mountain (adapted from White et al. 2023).

**Nature contact**

„incidental exposure such as residential proximity, relatively passive exposures such as nature documentaries, active engagement such as recreational visits and gardening, and interventions that use nature to promote health and well-being such as ‘green care’ and nature-based social prescribing initiatives.” (White et al. 2023)

**Nature-based therapy**

Planned therapeutic techniques performed in natural settings and based on nature–human active participation and connection (Adapted from Harper and Dobud 2020).

**Nature-based social prescribing**

A specific aspect of social prescribing, i.e. non-medical community referral approaches to connect individuals with community resources to support health and well-being, that aims to address health and wellbeing by connecting people with nature-based activities and experiences that require active participation, are socially supported, and generate meaning through these engagements (Leavell et al., 2019).

**Nature-based solutions**

"actions to protect, conserve, restore, sustainably use and manage natural or modified terrestrial, freshwater, coastal and marine ecosystems which address social, economic and environmental challenges effectively and adaptively, while simultaneously providing human well-being, ecosystem services, resilience and biodiversity benefits" (UNEP 2022)

→ see also Ecosystem Services, Resilience and Well-being

**Nature’s contribution to people**

“Nature’s contributions to people are all the contributions, both positive and negative, of living nature (i.e. diversity of organisms, ecosystems, and their associated ecological and evolutionary processes) to the quality of life for people. Beneficial contributions from nature include such things as food provision, water purification, flood control, and artistic inspiration, whereas detrimental contributions include disease transmission and predation that damages people or their assets. Many NCP may be perceived as benefits or detriments depending on the cultural, temporal or spatial context." (Díaz et al. 2018)

→ see also Ecosystem Services

**Resilience**

A collection, or stock, of adaptive resources that can be deployed to mitigate stress and persist disturbance. These resources and the processes through which they are deployed provide the capacity to adapt to change and persist disturbance by learning, self-organising, and transforming, while sustaining main processes, functions, and structure (Graham & Oswald, 2010; Folke et al. 2010).

**Biological resilience**

Adaptive, biological resources that contribute to resilience, for example a healthy immune system (Davydov et al. 2010).

**Biopsychosocial resilience**

Capacity resulting from combined biological, psychological, and social resilience to respond to various stressors (Davydov et al., 2010, White et al. 2023.)

**Community resilience**

The ability of a community, intended as a group of actors (people/individuals and organizations) linked by social ties, to cope with and recover from shocks, stresses and disturbances (Walker et al., 2004), including various types of socio-ecological challenges. It can be positively influenced by adaptability and transformability. In the context of RESONATE, the concept is understood as the result of successfully bidirectional interactions that occur at various levels between stocks of socio-economical and institutional resilience resources (social networks, governance structures, employments, laws) and stocks of socio-ecological resilience resources (land, water and other natural elements, i.e. available assets of all abiotic and biotic natural resources, including those man-made or managed by humans, such as landscapes) that, moving from individuals’ bio-psychosocial resilience (micro-level), by involving multiple actors and sectors in promoting and implementing effective Nature-based Therapies in specific contexts, brings direct positive effects on a larger group of actors, indirect positive benefits to the groups these actors belong to (e.g., families, associations), and the environments where they have interactions (meso-level).

→ see also Community

**Preventive resilience**

Reduction or mitigation of exposure to stressors before they result in disturbance of a system (WHO, 2020).

**Psychological resilience**

Adaptive, psychological resources that contribute to resilience, for example, optimism (Davydov et al. 2010).

**Recovery resilience**

Enhanced and/or more complete recovery or improved equilibrium following a reaction to a stressor or disturbance (White et al. 2023).

**Response resilience**

Adequate and flexible reaction to a stressor or disturbance (White et al. 2023)

**Social resilience**

Adaptive, social resources that contribute to resilience, for example, dispositional empathy (Davydov et al. 2010).

**Social-ecological resilience**

The capacity of a social-ecological system to absorb or withstand perturbations and other stressors such that the system remains within the same regime, essentially maintaining its structure and functions. It describes the degree to which the system is capable of self-organization, learning and adaptation. (Walker et al. 2004)

**Resilience hubs**

Cross-sectoral, multi-disciplinary, community- focused, physical (indoor or outdoor) and/or virtual creative space for designing, activating and maintaining social innovation actions based on continuous dialoguing of individuals and organizations involved within the local community about Nature-based Therapies, that seeks at supporting long-term resilience. In the context of RESONATE, it is understood as a network of actors (individuals and organizations) that engage in the design, development, test, implementation and maintenance of various types of activities, including events, seminars, experiential sessions, demonstrations, conferences, workshops, preparation of informative material and more, with the aim of promoting effective Nature-based Therapies and making them available to individuals, organizations and groups within their communities, thus contributing to community resilience.

→ see also Resilience, Community

**Social-ecological system**

“An integrated system that includes human societies and ecosystems, in which humans are part of nature. The functions of such a system arise from the interactions and interdependence of the social and ecological subsystems. The system’s structure is characterised by reciprocal feedbacks, emphasising that humans must be seen as a part of, not apart from, nature.” (IPCC 2022)

**Social innovation**

Reconfiguring of social practices, in response to societal challenges, which seeks to enhance outcomes on societal well-being and necessarily includes the engagement of civil society actors (Polman et al., 2017).

**Social innovation action (SIA)**

SIA constitutes a recent approach for hands-on practice and implementation of research and consolidating science-practice-policy links. It includes the organization of periodic networking events to encourage interested actors to present, learn about, discuss and initiate innovative inter-sectorial actions; the creation of market places (physical and virtual) for social innovation projects; and the start up or establishment of new networks amongst local actors. In the context of RESONATE, the concepts refer to possible social innovation processes that will be triggered by the Resilience Hubs and the actions that will be undertaken by stakeholders in these three locations with the aim of promoting Nature-based Therapies and, ultimately, increasing their and their communities' resilience.

→ see also Stakeholders, Community resilience

**Stakeholders**

Everyone who is directly or indirectly affected by or have a direct or indirect influence on a certain initiative, process, program or project (adapted from Rietbergen-McCracken and Narayan, 1998). It refers to individuals, organisations, and/or their associations and networks, differently structured and formalised; private, public or hybrid. Depending on size and level of action along the institutional and administrative scales, they can be international, regional-European, national, sub-national, provincial, or local-level stakeholders. In the context of RESONATE, stakeholders are individuals, organisations or networks affected by or able to influence the design, development, implementation, and scaling of Nature-based Therapies. They belong to two main groups, i.e. health/social/education-related and green areas/nature management/land planning-rela- ted sectors, even if others (e.g., media) can be relevant. New networks can promote social innovation actions and be results of social innovation processes.

→ see also Social network analysis, Social innovation action

**Stress**

“a threat, real or implied, to the psychological or physiological integrity of an individual.” (McEwen, 2000)

**Stress response**

Physiological and behavioral changes in response to exposure to stressors (McEwen & Stellar, 1993)

**Stressor**

Physical or psychological stimuli with the potential of disrupting homeostasis (Dickerson & Kemeny, 2004)

**Social network analysis**

A process to identify and map the reciprocal relationships and influences among actors (individuals or organisations-entities) connected in formal and informal networks in relation to a certain process/program/project design, implementation or evaluation. SNA allows typically to explore the existence and changes (in the time, or conditions) in the actors (nodes of a network) and in their relationships, and provides numerical indexes that can be a proxy of social-related concepts such as social capital, institutional trust or multilevel governance structures that are considered fundamental for achieving successful outcomes from social-economic related process, program or projects (Pisani et al. 2020). In the context of RESONATE, SNA is used to observe the networks among stakeholders in the Resilience Hubs and their local communities.

→ see also Resilience Hubs, Stakeholders

**Social acceptability**

It is a dynamic process in which subjects (individuals or groups) make judgements about the object of acceptance based on personal values, attitudes and intrapersonal perceptions (e.g., risks, etc.) and also through interpersonal interactions and communication, all being shaped by political, economic, and cultural context (Lucke 1995). Therefore, it is influenced by the networks among individuals and organizations interested on Nature-based Therapies (stakeholders), where information and other resources are exchanged. It can bring new values into a community, and therefore being part of a social innovation. Social acceptability has many degrees and includes behavioural aspects as well. These are: (1) rejection, (2) low acceptance, 3) indifference, (4) high acceptance, (5) proactive support, and (6) active ownership /adoption. Social acceptance is a positive result of a judgement process about an acceptance object or an object being considered as adequate, valid, and suitable (to a certain degree) by acceptance subjects compared to alternatives. We differentiate among socio-political, community and market acceptance.

→ see also Stakeholders, Social innovation

**Social awareness**

It is a cognitive component that can be described as knowledge, education, prior experience and/or understanding about an object by a subject. Social awareness relates to collective and social level awareness about a phenomenon. In the context of RESONATE, it can be raised by communicating a certain idea (narrative building), it characterises the actors involved (stakeholders and the whole local community) and therefore it is a key component of social innovation promoting Nature-based Therapies (based on Secco et al. 2017).

→ see also Stakeholders, Social innovation

**Quality of Life**

An “individual's perception of their position in life in the context of the culture and value systems in which they live and in relation to their goals, expectations, standards and concerns” (WHO 1997).

**Health related quality of life (HRQoL)**

“an individual's satisfaction or happiness with domains of life insofar as they affect or are affected by health” (Wilson and Cleary 1995)

**Well-being**

“The extent to which individuals have the ability to live the kinds of lives they have reason to value; the opportunities people have to achieve their aspirations. Basic components of human well-being include: security, material needs, health and social relations.” (MA 2005)

**References**

Davydov, D. M., Stewart, R., Ritchie, K., & Chaudieu, I. (2010). Resilience and mental health. *Clinical psychology review*, *30*(5), 479–495. <https://doi.org/10.1016/j.cpr.2010.03.003>

Díaz, S., Demissew, S., Carabias, J., Joly, C., Lonsdale, M., Ash, N., Larigauderie, A., Adhikari, J. R., Arico, S., Báldi, A., Bartuska, A., Baste, I. A., Bilgin, A., Brondizio, E., Chan, K. M. A., Figueroa, V. E., Duraiappah, A., Fischer, M., Hill, R., … Zlatanova, D. (2015). The IPBES Conceptual Framework — connecting nature and people. *Current Opinion in Environmental Sustainability*, *14*, 1–16. <https://doi.org/10.1016/J.COSUST.2014.11.002>

Dickerson, S. S., & Kemeny, M. E. (2004). Acute stressors and cortisol responses: A theoretical integration and synthesis of laboratory research. *Psychological Bulletin*, 130, 355–391.

EEA. https://www.eea.europa.eu/themes/sustainability-transitions/urban-environment/urban-green-infrastructure/glossary-for-urban-green-infrastructure

FAO. (2020). *Global Forest Resources Assessment 2020: Main Report*. <https://doi.org/https://doi.org/10.4060/ca9825en>

Folke Carpenter, S.R., Walker, B., Scheffer, M., Chapin, T., Rockström, J., C. (2010). Resilience thinking: integrating resilience, adaptability and transformability. *Ecology and Society*, *15*(4), 20.

Graham, L., & Oswald, A. J. (2010). Hedonic capital, adaptation and resilience. Journal of Economic Behavior & Organization, 76(2), 372-384. [https://doi.org/10.1016/j.jebo.2010.07.003](https://psycnet.apa.org/doi/10.1016/j.jebo.2010.07.003)

Grellier, J., White, M. P., Albin, M., ..., Nieuwenhuijsen, M. J., van den Bosch, M., ..., & Fleming, L. E. (2017). BlueHealth: a study programme protocol for mapping and quantifying the potential benefits to public health and well-being from Europe’s blue spaces. *BMJ Open*, *7*(6), e016188. <https://doi.org/10.1136/bmjopen-2017-016188>

Harper, N., Dobud, W. (2020). *Outdoor Therapies: An Introduction to Practices, Possibilities, and Critical Perspectives*. October 2020. Publisher: Routledge, Taylor & Francis GroupISBN: 9780367365707

IPCC. (2022). *Climate Change 2022: Impacts, Adaptation, and Vulnerability. Contribution of Working Group II to the Sixth Assessment Report of the Intergovernmental Panel on Climate Change* (H.-O. Pörtner  Roberts, D.C:, Tignor, M., Poloczanska, E.S., Mintenbeck, K., Alegría, A., Craig, M., Langsdorf, S., Löschke, S., Möller, V., Okem, A., Rama, B., Ed.).

Lucke D (1995) Akzeptanz: Legitimitat in der «Abstimmungsgesellschaft». VS Verlag für Sozialwissenschaften, Wiesbaden. https://doi.org/10.1007/978-3-663-09234-6

Konijnendijk, C., Devkota, D., Mansourian, S. & Wildburger, C. (eds.), 2023. Forests and Trees for Human Health: Pathways, Impacts, Challenges and Response Options. A Global Assessment Report. IUFRO World Series Volume 41. Vienna. 232 p.

Leavell, M. A., Leiferman, J. A., Gascon, M., Braddick, F., Gonzalez, J. C., & Litt, J. S. (2019). Nature-Based Social Prescribing in Urban Settings to Improve Social Connectedness and Mental Well-being: a Review. *Current Environmental Health Reports*. https://doi.org/10.1007/s40572-019-00251-7

MA. (2005). *Millennium ecosystem assessment: Ecosystems and Human Well-Being*. Island Press. https://www.millenniumassessment.org/documents/document.356.aspx.pdf

MacQueen, K. M., McLellan, E., Metzger, D. S., Kegeles, S., Strauss, R. P., Scotti, R., Blanchard, L., & Trotter, R. T., 2nd (2001). What is community? An evidence-based definition for participatory public health. *American journal of public health*, *91*(12), 1929–1938. <https://doi.org/10.2105/ajph.91.12.1929>

McEwen, B., Stellar, E., B. (1993). Stress and the individual: Mechanisms leading to disease. *Archives of Internal Medicine*, *153*(18), 2093–2101. <https://doi.org/10.1001/archinte.1993.00410180039004>

McEwen, B. S. (1998). Stress, Adaptation, and Disease: Allostasis and Allostatic Load. *Annals of the New York Academy of Sciences*, *840*(1), 33–44. <https://doi.org/10.1111/j.1749-6632.1998.tb09546.x>

McEwen, B. S. (2000). The neurobiology of stress: From serendipity to clinical relevance. *Brain Research*, 886(1–2), 172–189. https://doi.org/10.1016/S0006-8993(00)02950-4

McMillan, D.W. and Chavis, D.M. (1986), Sense of community: A definition and theory. J. Community Psychol., 14: 6-23. [https://doi.org/10.1002/1520-6629(198601)14:1<6::AID-JCOP2290140103>3.0.CO;2-I](https://doi.org/10.1002/1520-6629(198601)14:1%3C6::AID-JCOP2290140103%3E3.0.CO;2-I)

Miller, R.W. (1988). Urban Forestry: Planning and Managing Urban Greenspaces.

Pisani, E., Laidin, C., Masiero, M., Secco, L., & Pettenella, D. (2020). 41. Project networks funded by LEADER across Europe–a proposed evaluating approach based on social network analysis. In Green metamorphoses: agriculture, food, ecology (pp. 443-450). Wageningen Academic.

Polman, N., Slee, B., Kluvánková, T., Dijkshoorn-Dekker, M., Nijnik, M., Gezik, V., & Soma, K. (2017). Classification of social innovations for marginalised rural areas (Deliverable D2.1). Social Innovation in Marginalised Rural Areas (SIMRA) Project, Horizon 2020 (Grant Agreement No. 677622). European Commission. <https://www.simra-h2020.eu/wp-content/uploads/2017/09/SIMRA-D2.1.pdf>

Secco, L., Pisani, E., Burlando, C., Da Re, R., Gatto, P., Pettenella, D., Vassilopoulus, A., Akinsete, E., Koundouri, P., Lopolito, A., Prosperi, M., Tuomasiukka, D., Den Herde, M., Lovric, M., Polman, N., Dijkshoorn, M., Soma, K., Ludvig, A., Weiss, G., Zivojinovic, I., Sarkki, S., Ravazzoli, E., Dalla Torre, C., Streifeneder, T., Slee, B., Nijnik, M., Miller, D., Barlagne, C. and Prokofieva, I. 2017. Set of methods to assess SI implications at different levels: instructions for WPs 5&6, Deliverable D4.2, Social Innovation in Marginalized Rural Areas Project (SIMRA), Demonstrator to the European Commission, pp.203.

Sterling, P., & Eyer, J. (1988). Allostasis: a new paradigm to explain arousal pathology. In S. R. J. Fisher (Ed.), *Handbook of life stress, cognition and health* (pp. 629–649). John Wiley & Sons.

UNEP (2022). Nature-based Solutions for Supporting Sustainable Development (UNEA Resolution 5/5). United Nations Environment Assembly of the United Nations Environment Programme, Nairobi. Available at: <https://wedocs.unep.org/handle/20.500.11822/39864>

White, M. P., Hartig, T., Martin, L., Pahl, S., van den Berg, A. E., Wells, N. M., Costongs, C., Dzhambov, A. M., Elliott, L. R., Godfrey, A., Hartl, A., Konijnendijk, C., Litt, J. S., Lovell, R., Lymeus, F., O’Driscoll, C., Pichler, C., Pouso, S., Razani, N., … van den Bosch, M. (2023). Nature-based biopsychosocial resilience: An integrative theoretical framework for research on nature and health. *Environment International*, *181*, 108234. https://doi.org/https://doi.org/10.1016/j.envint.2023.108234

Walker, B., C. S. Holling, S. R. Carpenter, and A. Kinzig. 2004. Resilience, adaptability and transformability in social–ecological systems. Ecology and Society 9(2): 5.

WHO. (1948). *Preamble to the Constitution of the World Health Organization as adopted by the International Health Conference, New York, 19-22 June, 1946; signed on 22 July 1946 by the representatives of 61 States (Official Records of the World Health Organization)*

World Health Organization. (1997). WHOQOL: Measuring quality of life. Geneva: World Health Organization. <https://www.who.int/tools/whoqol>

World Health Organization. (2020). *Operational framework for building climate resilient health systems.* Geneva: World Health Organization. https://www.who.int/publications/i/item/9789240019039

Wilson, I. B., & Cleary, P. D. (1995). Linking clinical variables with health-related quality of life: A conceptual model of patient outcomes. JAMA: The Journal of the American Medical Association, 273(1), 59–65. <https://doi.org/10.1001/jama.1995.03520250075037>
