# Supplementary material for: Testing nature-based biopsychosocial resilience theory: a research programme protocol
Source: Arch Public Health. 2026 Apr 24;84:90. doi: 10.1186/s13690-026-01903-5 (PMC13107860; doi:10.1186/s13690-026-01903-5)
Supplement: Supplementary file 2 — Supplementary Material 2. [file 13690_2026_1903_MOESM2_ESM.docx]

**Appendix A: Abridged RESONATE Glossary**

The full Glossary containing extended definitions of many of the terms below that are used by consortium members and all terms discussed and agreed upon during the Delphi process, including those not presented here, is presented in the Supplementary Materials. Further sources and references are also presented in the Supplementary Materials.

| **Nature**  ***Blue space***: “outdoor environments - either natural or manmade - that prominently feature water” (Grellier et al. 2017) (e.g., rivers, lakes, ponds, canals, fountains, coastal margins). **→ see also Green space**  ***Blue infrastructure***: “Blue infrastructure includes bodies of water, watercourses, ponds, lakes and storm drainage, that provide ecological and hydrological functions including evaporation, transpiration, drainage, infiltration and temporarily storage of runoff and discharge.” (IPCC 2022) → see also Green infrastructure  ***Green infrastructure***: “The strategically planned interconnected set of natural and constructed ecological systems, green spaces and other landscape features that can provide functions and services including air and water purification, temperature management, floodwater management and coastal defence often with co-benefits for human and ecological well-being. Green infrastructure includes planted and remnant native vegetation, soils, wetlands, parks and green open spaces, as well as building and street-level design interventions that incorporate vegetation.” (IPCC 2022) → see also Blue infrastructure  ***Green space***: Outdoor environments - either natural or man- made - that prominently feature vegetation. **→ see also Blue space**  ***Nature***: Nature “refers to the natural world with an emphasis on its living components. Within the context of western science, it includes categories such as biodiversity, ecosystems (both structure and functioning), evolution, the biosphere; humankind’s shared evolutionary heritage, and biocultural diversity. Within the context of other knowledge systems, it includes categories such as Mother Earth and systems of life, and it is often viewed as inextricably linked to humans, not as a separate entity.” (IPBES, 2020)  ***Nature contact***: “incidental exposure such as residential proximity, relatively passive exposures such as nature documentaries, active engagement such as recreational visits and gardening, and interventions that use nature to promote health and well-being such as ‘green care’ and nature-based social prescribing initiatives.” (White et al. 2023)  **Nature-based interventions**  ***Nature-based therapy***: Planned therapeutic techniques performed in natural settings and based on nature–human active participation and connection (Adapted from Harper and Dobund 2021).  *Nature-based social prescribing*: A specific aspect of social prescribing, i.e., non-medical community referral approaches to connect individuals with community resources to support health and well-being, that aims to address health and wellbeing by connecting people with nature-based activities and experiences that require active participation, are socially supported, and generate meaning through these engagements (Leavell et al., 2019).  ***Nature-based solutions***: "actions to protect, conserve, restore, sustainably use and manage natural or modified terrestrial, freshwater, coastal and marine ecosystems which address social, economic and environmental challenges effectively and adaptively, while simultaneously providing human well-being, ecosystem services, resilience and biodiversity benefits" (UNEP 2022) → see also Ecosystem Services, Resilience and Well-being  **Health/Well-being**  ***Health***: “a state of complete physical, mental and social well-being and not merely the absence of disease or infirmity” (WHO 1984)  ***Health related quality of life (HRQoL)***: “an individual's satisfaction or happiness with domains of life insofar as they affect or are affected by health” (Wilson & Cleary 1995)  ***Homeostasis***: “a self-regulating process by which biological systems maintain stability while adjusting to changing external conditions. This concept explains how an organism can maintain more or less constant internal conditions that allow it to adapt and to survive in the face of a changing [...] external environment.” (Billman 2020)  ***Quality of Life***: An “individual's perception of their position in life in the context of the culture and value systems in which they live and in relation to their goals, expectations, standards and concerns” (WHO 2012).  ***Stress***: “a threat, real or implied, to the psychological or physiological integrity of an individual.” (McEwen, 2000)  ***Stress response***: “physiological and behavioral changes in response to exposure to stressors” (Chu et al 2022)  ***Stressor***: Physical or psychological stimuli with the potential of disrupting homeostasis (Chu et al 2022)  ***Well-being***: “The extent to which individuals have the ability to live the kinds of lives they have reason to value; the opportunities people have to achieve their aspirations. Basic components of human well-being include: security, material needs, health and social relations.” (UNEP 2007)  **Resilience**  ***Resilience***: A collection, or stock, of adaptive resources that can be deployed to mitigate stress and persist disturbance. These resources and the processes through which they are deployed provide the capacity to adapt to change and persist disturbance by learning, self-organising, and transforming, while sustaining main processes, functions, and structure (Folke et al. 2010).  ***Biological resilience***: Adaptive, biological resources that contribute to resilience, for example a healthy immune system (Davydov et al. 2010).  ***Biopsychosocial resilience***: Capacity resulting from combined biological, psychological, and social resilience to respond to various stressors (Davydov et al., 2010).  ***Community resilience***: The ability of a community, intended as a group of actors (people/individuals and organizations) linked by social ties, to cope with and recover from shocks, stresses and disturbances (Walker et al., 2004), including various types of socio-ecological challenges. → see also Community  ***Preventive resilience***: Reduction or mitigation of exposure to stressors before they result in disturbance of a system (WHO, 2020).  ***Psychological resilience***: Adaptive, psychological resources that contribute to resilience, for example, optimism (Davydov et al. 2010).  ***Recovery resilience***: Enhanced and/or more complete recovery or improved equilibrium following a reaction to a stressor or disturbance (White et al. 2023).  ***Resilience hubs***: Cross-sectoral, multi-disciplinary, community- focused, physical (indoor or outdoor) and/or virtual creative space for designing, activating and maintaining social innovation actions based on continuous dialoguing of individuals and organizations involved within the local community about Nature-based Therapies, that seeks at supporting long-term resilience. → see also Resilience, Community  ***Response resilience***: Adequate and flexible reaction to a stressor or disturbance (White et al. 2023)  ***Social resilience***: Adaptive, social resources that contribute to resilience, for example, dispositional empathy (Davydov et al. 2010).  ***Social-ecological resilience***: “the capacity of a social-ecological system to absorb or withstand perturbations and other stressors such that the system remains within the same regime, essentially maintaining its structure and functions. It describes the degree to which the system is capable of self-organization, learning and adaptation.” (Resilience Alliance, n.d.)  **Society/Community**  ***Community***: A group of people who are linked by social ties, share common interests, values, and engage in social interactions to fulfill mutual needs and aspirations in geographical locations or settings (based on MacQueen et al., 2001). → see also Community resilience  ***Equity***: “The principle of being fair and impartial, and a basis for understanding how the impacts and responses to threats and opportunities, including costs and benefits, are distributed in and by society in more or less equal ways. Often aligned with ideas of equality, fairness and justice and applied with respect to equity in the responsibility for, and distribution of, policies across society, generations and gender, and in the sense of who participates and controls the processes of decision-making.” (IPCC 2022)  ***Social acceptability***: It is a dynamic process in which subjects (individuals or groups) make judgements about the object of acceptance based on personal values, attitudes and intrapersonal perceptions (e.g., risks, etc.) and also through interpersonal interactions and communication, all being shaped by political, economic, and cultural context (Wolsink 2018). → see also Stakeholders, Social innovation  ***Social-ecological system***: “An integrated system that includes human societies and ecosystems, in which humans are part of nature. The functions of such a system arise from the interactions and interdependence of the social and ecological subsystems. The system’s structure is characterised by reciprocal feedbacks, emphasising that humans must be seen as a part of, not apart from, nature.” (IPCC 2022)  ***Social innovation action***: SIA constitutes a recent approach for hands-on practice and implementation of research and consolidating science-practice-policy links. In the context of RESONATE, the concepts refer to possible social innovation processes that will be triggered by the Resilience Hubs and the actions that will be undertaken by stakeholders in these three locations with the aim of promoting Nature-based Therapies and, ultimately, increasing their and their communities' resilience. → see also Stakeholders, Community resilience  ***Stakeholders***: Everyone who is directly or indirectly affected by or have a direct or indirect influence on a certain initiative, process, program or project (adapted from Rietbergen-McCracken and Narayan, 1998). → see also, Social innovation action |
| --- |
